# Digital Storytelling Intervention for Hemoglobin A_1c_ Control Among Hispanic Adults With Type 2 Diabetes: A Randomized Clinical Trial

**DOI:** 10.1001/jamanetworkopen.2024.24781

**Published:** 2024-08-02

**Authors:** Mark L. Wieland, Katherine Diaz Vickery, Valentina Hernandez, Becky R. Ford, Crystal Gonzalez, Silvio Kavistan, Sheila Iteghete, Christi A. Patten, Jane W. Njeru, Abby M. Lohr, Jamie O’Byrne, Paul J. Novotny, Davinder P. Singh, Linda K. Larkey, Miriam Goodson, Graciela Porraz Capetillo, Irene G. Sia

**Affiliations:** 1 Division of Community Internal Medicine, Geriatrics, and Palliative Care, Mayo Clinic, Rochester, Minnesota; 2 Health, Homelessness & Criminal Justice Lab, Hennepin Healthcare Research Institute, Minneapolis, Minnesota; 3 Mountain Park Health Centfer, Phoenix, Arizona; 4 Community Based Research, Mayo Clinic, Rochester, Minnesota; 5 Department of Psychiatry and Psychology, Mayo Clinic, Rochester, Minnesota; 6 Division of Epidemiology, Mayo Clinic, Rochester, Minnesota; 7 Division of Biomedical Statistics and Informatics, Mayo Clinic, Rochester, Minnesota; 8 Edson College of Nursing and Health Innovation, Arizona State University, Phoenix; 9 Alliance of Chicanos, Hispanics, and Latin Americans, Rochester, Minnesota; 10 Division of Public Health, Infectious Diseases, and Occupational Medicine, Mayo Clinic, Rochester, Minnesota

## Abstract

**Importance:**

Hispanic adults with type 2 diabetes (T2D) are more likely to develop complications and die from the disease than the US general population. Digital storytelling interventions are narrative-based videos elicited through a community-based participatory research approach to surface the authentic voices of participants overcoming obstacles to health-promoting behaviors that perpetuate health inequities; research on the effect of digital storytelling on T2D outcomes among Hispanic adults is lacking.

**Objective:**

To assess the impact of a digital storytelling intervention on glycemic control and its acceptability among Hispanic patients with poorly controlled T2D.

**Design, Setting, and Participants:**

This was a multicenter, randomized clinical trial conducted within 2 primary care networks in Minnesota and Arizona among Hispanic adults with poorly controlled T2D (hemoglobin A_1c_ level ≥8%). Enrollment and follow-up were conducted between February 14, 2019, and November 1, 2023.

**Intervention:**

The intervention group viewed a 12-minute digital storytelling video. The video included 4 Spanish-language stories that reinforced 4 diabetes self-management behavioral goals (healthful diet for diabetes, physical activity, medication adherence, and glucose self-monitoring). The control group received printed, culturally tailored T2D education materials.

**Main Outcomes and Measures:**

The primary outcome was the mean change from baseline to 3 months for hemoglobin A_1c_ levels, adjusting for baseline hemoglobin A_1c_, age, gender, education, and income. Acceptability and narrative quality of the intervention were assessed through questionnaires.

**Results:**

There were 451 study participants, with 227 (mean [SD] age, 54.3 [9.3] years; 158 [69.3%] women) randomized to the intervention group and 224 (mean [SD] age, 54.5 [9.1] years; 156 [69.3%] women) to the control group. Of these, 390 completed 3-month follow-up of the primary outcome (86% retention). There was a small improvement in the mean (SD) hemoglobin A_1c_ level in the intervention group compared with the control group in the adjusted model (9.1% [1.7] to 8.4% [1.6] vs 9.4% [1.8] to 8.8% [2.0]; *P* = .04] but not in the unadjusted model. Acceptability and narrative quality of the intervention were high.

**Conclusions and Relevance:**

In this randomized clinical trial, a digital storytelling intervention developed with and for Hispanic adults with T2D was highly acceptable and feasibly implemented within primary care settings and resulted in a modest improvement of glycemic control. This was a highly scalable intervention that may be integrated into clinical practice as part of a longitudinal diabetes self-management program for Hispanic adults.

**Trial Registration:**

ClinicalTrials.gov Identifier: NCT03766438

## Key Points

**Question:**

What are the acceptability, feasibility, and effectiveness of a digital storytelling intervention for type 2 diabetes self-management among Hispanic adults in primary care settings?

**Findings:**

In this randomized clinical trial with 451 participants, Hispanic patients who received a digital storytelling intervention in primary care settings had a small improvement in glycemic control at 3 months compared with patients who did not receive the intervention. The intervention was highly acceptable and feasibly implemented.

**Meaning:**

This is a highly scalable intervention that may be integrated into clinical and public health practice as part of a longitudinal diabetes self-management program for Hispanic adults.

## Teaser

This randomized clinical trial tests whether a digital storytelling intervention improves glycemic control among Hispanic adults with type 2 diabetes (T2D).

## Introduction

Hispanic adults in the United States with type 2 diabetes (T2D) are more likely to develop complications and die from the disease than the general population.^1^ These disparities are compounded by a 1.6 times higher age-adjusted T2D prevalence among Hispanic adults compared with non-Hispanic White adults.^2^ The majority of evidence-based T2D interventions have been developed and tested within predominantly non-Hispanic White populations and often are implemented within Hispanic populations without consideration of the need for cultural grounding or adaptation. Previous research has demonstrated improved outcomes of conventional T2D clinic- and community-based interventions when culturally tailored for Hispanic populations.^3^

Narrative-based (storytelling) interventions are a series of promising approaches that incorporate culture-centric health messaging to promote behavior change.^4^ Storytelling interventions use narratives that resonate with target populations either through direct quotes from representative members or through story compositions inspired by culturally embedded informants.^5^ The ways in which stories elicit behavioral responses have been conceptualized as identification and transportation.^6,7^ Identification with the storytellers is an important step for engagement, empowerment, and reframing social norms.^8,9,10^ Transportation of the listener into the story is likewise important to generate persuasive effects for behavior change.^11^ This approach is especially promising in populations with a strong oral tradition,^12^ including those from Latin America.

Narrative-based video interventions, where story components are incorporated into health communication media, provide the opportunity for wide distribution and inclusion of consistent content to promote health behavior change. In the first randomized trial of a storytelling video intervention for chronic disease management of which we are aware, Houston and colleagues^13^ demonstrated efficacy comparable with adding a medication for treatment of uncontrolled hypertension among African American viewers. Campbell and colleagues^14^ established the promising role of storytelling videos in management of diabetes through a randomized trial among patients with T2D in Australia, which demonstrated improvements in self-reported diabetes self-management among viewers.

Digital stories are narrative-based videos elicited through a community-based participatory research (CBPR)^15^ approach to surface the authentic voices of participants overcoming obstacles to health-promoting behaviors.^16^ They differ from other forms of narrative-based videos in that participants are central to the production of knowledge. Through a group-based digital storytelling workshop, storytellers build their own narrative, choose images and sounds that best represent their experiences, and are guided through editing.^17^ Through a process of social construction, individuals and groups derive concepts and actions to co-create meaning. Participants construct their own experiences in a group setting of peers who, through reaction and feedback, contribute in turn to the shared understanding of the individuals’ experiences.^17^ This differs from the communication paradigm in which experts generalize an experience for a community. The process of developing digital storytelling interventions has been used to empower participants through personal reflection^18,19,20^ and as a tool for health advocacy,^21^ but the resultant videos can also shape health behaviors of viewers by influencing attitudes and beliefs.^22^ In this way, digital storytelling interventions represent a highly scalable approach that can be rapidly incorporated into clinical and public health practice.

A digital storytelling intervention for T2D self-management (using the digital storytelling workshop procedures to create videos) was developed by Rochester Healthy Community Partnership (RHCP) to address 4 core behavioral goals of T2D self-management: healthful diabetes diet, physical activity, medication adherence, and glucose self-monitoring.^23^ RHCP is a 20-year CBPR partnership that is productive and experienced at deploying interventions and evaluation with immigrant populations.^24^ RHCP community partners identified T2D as a priority area for investigation, selected the digital storytelling approach, and participated in all formative work and intervention development.^25^ Formative assessment with clinical partners demonstrated preliminary evidence for feasibility and acceptability of the intervention.^26^ This study presents the results of a randomized clinical trial to assess the impact of the digital storytelling intervention on glycemic control among Hispanic patients with poorly controlled T2D and to assess the acceptability of the intervention among a large study population within primary care settings.

## Methods

### Trial Design and Participants

This study evaluated the digital storytelling intervention through a 2-group, parallel randomized clinical trial in clinical settings across 2 health care institutions among Hispanic adults with poorly controlled T2D (hemoglobin A_1c_ level ≥8% [to convert to proportion of total hemoglobin, multiply by 0.01]). The intervention group viewed the 12-minute digital storytelling intervention in addition to receiving usual clinical care. The comparison group received usual clinical care. Both groups received culturally tailored T2D self-management education materials. The study logic model is presented in eFigure 1 in Supplement 1. The primary outcome was glycemic control as measured by hemoglobin A_1c_ level. The study design is in accordance with the Consolidated Standards of Reporting Trials (CONSORT) statement for reporting parallel group randomized trials,^27^ and the protocol (Supplement 2) has previously been reported and registered with the Clinical Trials Registry (NCT03766438). All study procedures were approved by the Mayo Clinic Institutional Review Board. All participants provided written informed consent.

The clinical trial was conducted at 2 primary care networks with large proportions of Hispanic patients in Arizona and Minnesota. Practice characteristics are described in the eMethods in Supplement 1. Eligibility criteria included (1) self-identify as Hispanic or Latino, (2) age 18 to 70 years, (3) receive primary care at 1 of the participating clinical sites, (4) at least 1 office visit within the previous 12 months, (5) diagnosis of T2D in the medical record, (6) T2D diagnosis for 6 months or longer, (7) most recent hemoglobin A_1c_ measurement 8% or higher, and (8) intention to continue to receive medical care at the recruitment clinic for the next 3 months. Only 1 member of a household was eligible. Recruitment and follow-up were conducted between February 14, 2019, and November 1, 2023.

### Screening and Group Assignment

Once eligible patients were identified from institutional registries, the next office appointment for any indication was identified. After a telephone screen, patients were sequentially recruited at their office visit until the target accrual was reached.

After obtaining consent, baseline measures were obtained, followed by group assignment by a software package. Permuted block randomization with blocks of 4 was used for assignment, with stratification according to site and gender. Data analysts were blinded to treatment condition.

### Intervention

Language- and culture-congruent study staff showed the 12-minute video to each participant in a private room. It included an introduction by an RHCP community partner, 4 Spanish-language stories, and a closing educational message reenforcing the 4 diabetes self-management behavioral goals. The storytellers included 2 women and 2 men, reflecting some of the heterogeneity among Hispanic subgroups in the United States (2 Mexican, 1 Central American, and 1 South American storyteller). To ensure that participants received and understood the video, study staff asked each participant 3 questions^28^ immediately after viewing: (1) What is your reaction to the video? (2) What was the main message of the video? and (3) Does the video motivate you to make any changes to the way you manage your diabetes?

Participants were provided access to the storytelling video as an application on their mobile phones and/or a DVD, flash drive, and web link. The software application was presented in Spanish or English (depending on the preferred language presets of participant’s mobile phone) and included the intervention with direct access to individual stories as well as culturally tailored educational material about each of the 4 T2D self-management goals. To increase the likelihood that participants watched the storytelling video throughout the study interval, participants received a monthly automated text message (5 total) that asked them to self-rate their motivational level and self-efficacy for managing T2D (0, indicating no motivation or self-confidence, to 10, indicating extremely motivated or confident) and recommended that they watch the intervention if they scored lower than 7. A link to the intervention and a quick response code for the software application are available in eMethods in Supplement 1.

### Control Condition

The control group received usual diabetes clinical care. They also received paper copies of the culturally tailored T2D education material.

### Data Collection, Outcome Measures, and Covariates

Participant data were collected at baseline and 3 months. Biometric measurements were obtained during clinic visits. Participants completed a survey to obtain demographic information and theory-based factor measurements. Additional clinical data were collected from electronic medical records.

The primary outcome measure was glycemic control as measured by hemoglobin A_1c_ from whole blood samples and analyzed by the clinical laboratories at each study site. During the COVID-19 pandemic, participants were given the option of home testing via a validated point-of-care hemoglobin A_1c_ test. The study team mailed the device to the participant, and a video call was scheduled to directly observe the test procedures and record the result. A total of 44 participants (7.3%) did home-based hemoglobin A_1c_ testing. Sensitivity analyses were performed by rerunning the models presented in the data analysis section by each hemoglobin A_1c_ sample subgroup (point-of-care vs venipuncture). There were no differences in the primary outcome between groups.

Secondary measures included blood pressure, low-density lipoprotein (LDL) cholesterol, body mass index (BMI; calculated as weight in kilograms divided by height in meters squared), and diabetes self-management behaviors.^29^ Seated blood pressure measurements were made on the right arm using an automated device after sitting quietly for 5 minutes; the average of 2 readings was used in analyses. Weight was measured to the nearest 0.1 kg using a digital scale. Height was measured to the nearest 0.1 cm using a stadiometer. Total cholesterol, high-density lipoprotein (HDL) cholesterol, and triglyceride levels were measured from the same blood sample. LDL cholesterol was calculated as total cholesterol − HDL cholesterol − (triglycerides/5). Diabetes self-management behaviors were assessed with the Summary of Diabetes Self-Care Activities Measure^30^ across the following domains: general diet, specific (diabetes) diet, physical activity, diabetes medication use, and blood glucose monitoring.

Participant demographic characteristics and diabetes-related comorbidities were assessed by survey items at baseline. The number of diabetes-related office visits for the 6-month interval before and after intervention (or control) delivery were abstracted from the electronic medical records.

For patients in the intervention group, acceptability was assessed using an adapted health communication assessment tool produced by the National Cancer Institute,^31^ including acceptability of the storytelling videos and the extent to which the videos captured their attention. Participants rated their confidence (self-efficacy) about managing diabetes as a result of watching the storytelling video on a 3-point Likert scale (much more confident, somewhat more confident, no more confident).^26^ Participants were asked whether the video motivated them to make any changes in their T2D self-management; those who replied yes were asked open-ended questions about any new behavioral intentions after watching the video, which were collated into a prepopulated list.

For patients in the intervention group, narrative quality of the intervention was assessed via the Narrative Quality Assessment Tool subcomponents of story identification and transportation through a 14-item instrument on a 5-point Likert scale (with 5 being the highest value) developed by Larkey and colleagues^32^ that demonstrated good construct validity among Hispanic, Spanish-speaking populations,^33^ with predictive validity on the transportation (emotional engagement) scale.^34^

### Statistical Analysis

Intervention acceptability and narrative quality were reported using descriptive statistics. The primary analysis was a comparison of hemoglobin A_1c_ levels between the intervention and control groups at 3 months compared with baseline, while secondary analyses included difference between groups in systolic and diastolic blood pressure, BMI, weight, and LDL cholesterol. All analyses were adjusted for baseline hemoglobin A_1c_ values only and covariance adjusted for age, gender, education, and household income (analysis of covariance [ANCOVA]) as important a priori effect modifiers.^29^ Estimates and 95% CIs of the adjusted mean change in each measure summarized this activity, and *P* values were generated for the unadjusted and adjusted ANCOVA analyses. A comparison between study groups on whether participants achieved a hemoglobin A_1c_ goal of less than 8% at 3 months was conducted using logistic regression on a dichotomized assessment of the 3-month hemoglobin A_1c_ value. Assessment of missing data are reported in the eMethods in Supplement 1.

## Results

A total of 451 participants were enrolled and randomized, with 227 (mean [SD] age, 54.3 [9.3] years; 158 [69.3%] women) randomized to the intervention group and 224 (mean [SD] age, 54.5 [9.1] years; 156 [69.3%] women) to the control group. Baseline characteristics are shown in Table 1. Participants had suboptimal glycemic control at baseline, with mean hemoglobin A_1c_ greater than 9% in both groups. The mean BMI was in the obesity range (>30), but baseline mean LDL cholesterol levels and blood pressure were within the reference range (Table 1). At 3 months, 390 participants completed measures (86% follow-up), with no significant difference on study retention between groups (intervention group, 191 [84.1%]; control group, 199 [88.8%]; *P* = .24) (Figure).

**Table 1.** Baseline Characteristics of the Study Sample

Characteristic	Participants, No. (%)	
	Intervention group (n = 227)	Control group (n = 224)
Age, mean (SD), y	54.3 (9.3)	54.5 (9.1)
Gender		
Women	158 (69.3)	156 (69.3)
Men	69 (30.7)	68 (30.7)
Education		
Eighth grade or less	110 (48.9)	105 (46.5)
Some high school	53 (23.6)	53 (23.5)
High school graduate or GED	35 (15.6)	39 (17.3)
Some college or technical school	17 (7.6)	22 (9.7)
College or graduate degree	10 (4.4)	7 (3.1)
Average family income, $		
0 to 9999	84 (39.4)	79 (38.7)
10 000 to 19 999	39 (18.3)	58 (28.4)
20 000 to 29 999	46 (21.6)	29 (14.2)
30 000 to 39 999	20 (9.4)	24 (11.8)
≥40 000	24 (11.3)	14 (6.9)
Work status		
Full time	62 (27.2)	66 (29.3)
Part time	53 (23.2)	42 (18.7)
Unemployed, retired, or disabled	113 (49.6)	117 (52.0)
Years living with diabetes, mean (SD)	12.7 (8.75)	12.2 (8.53)
Born in the United States	22 (9.7)	25 (11.1)
English language spoken at home	22 (9.6)	28 (12.3)
Self-rated English language speaking proficiency		
Not at all or not very well	177 (78.0)	168 (74.3)
Well or very well	50 (22.0)	58 (25.7)
Clinic site		
Mountain Park	107 (46.9)	107 (47.1)
Hennepin Healthcare	121 (53.1)	120 (52.9)
Health insurance		
No insurance	59 (26.0)	68 (30.5)
Medicaid or Medicare	81 (35.7)	75 (33.6)
Private insurance	21 (9.3)	23 (10.3)
Hemoglobin A_1c_, mean (SD), %	9.1 (1.72)	9.4 (1.75)
LDL cholesterol, mean (SD), mg/dL	98.6 (40.11)	99.3 (39.22)
Systolic BP, mean (SD), mm Hg	126.6 (16.48)	130.3 (19.22)
Diastolic BP, mean (SD), mm Hg	76.4 (8.99)	77.3 (9.26)
Body mass index, mean (SD)^a^	32.5 (7.55)	31.1 (6.16)
Diabetes self-management behaviors, mean (SD)^b^		
General diet	3.7 (2.14)	4.0 (2.03)
Diabetes-specific diet	3.9 (1.70)	3.9 (1.73)
Exercise	2.4 (2.37)	2.7 (2.47)
Blood glucose testing	4.3 (2.80)	4.2 (2.80)
Diabetes medications use	6.6 (1.40)	6.5 (1.39)
Complications and comorbidities		
Nephropathy (or chronic kidney disease)	20 (8.8)	15 (6.6)
Retinopathy	26 (11.4)	25 (11.0)
Neuropathy	38 (16.7)	29 (12.8)
Coronary artery disease	10 (4.4)	9 (4.0)
Cerebrovascular disease or history of stroke	4 (1.8)	8 (3.5)
Peripheral arterial disease	6 (2.6)	4 (1.8)
Infections (eg, foot infections)	5 (2.2)	5 (2.2)
Diabetes medications		
Insulin (any preparation)	139 (61.0)	116 (51.1)
Other medication for diabetes	204 (89.5)	214 (94.3)
Office visits in the last 12 mo		
Primary care clinician	225 (98.7)	224 (98.7)
Endocrinologist	18 (7.9)	14 (6.2)
Dietician	61 (26.8)	63 (27.8)
Diabetes educator	2 (0.9)	6 (2.6)
Clinical pharmacy	48 (21.1)	49 (21.6)
Other diabetes-related visits	26 (11.4)	28 (12.3)

Abbreviations: BP, blood pressure; LDL, low-density lipoprotein.

SI conversion factors: To convert hemoglobin A_1c_ to proportion of total hemoglobin, multiply by 0.01; LDL cholesterol to millimoles per liter, multiply by 0.0259.

^a^ Body mass index was calculated as weight in kilograms divided by height in meters squared.

^b^ Diabetes self-management behaviors were assessed by No. of d/wk participants (on average) met each goal.

**Figure.**
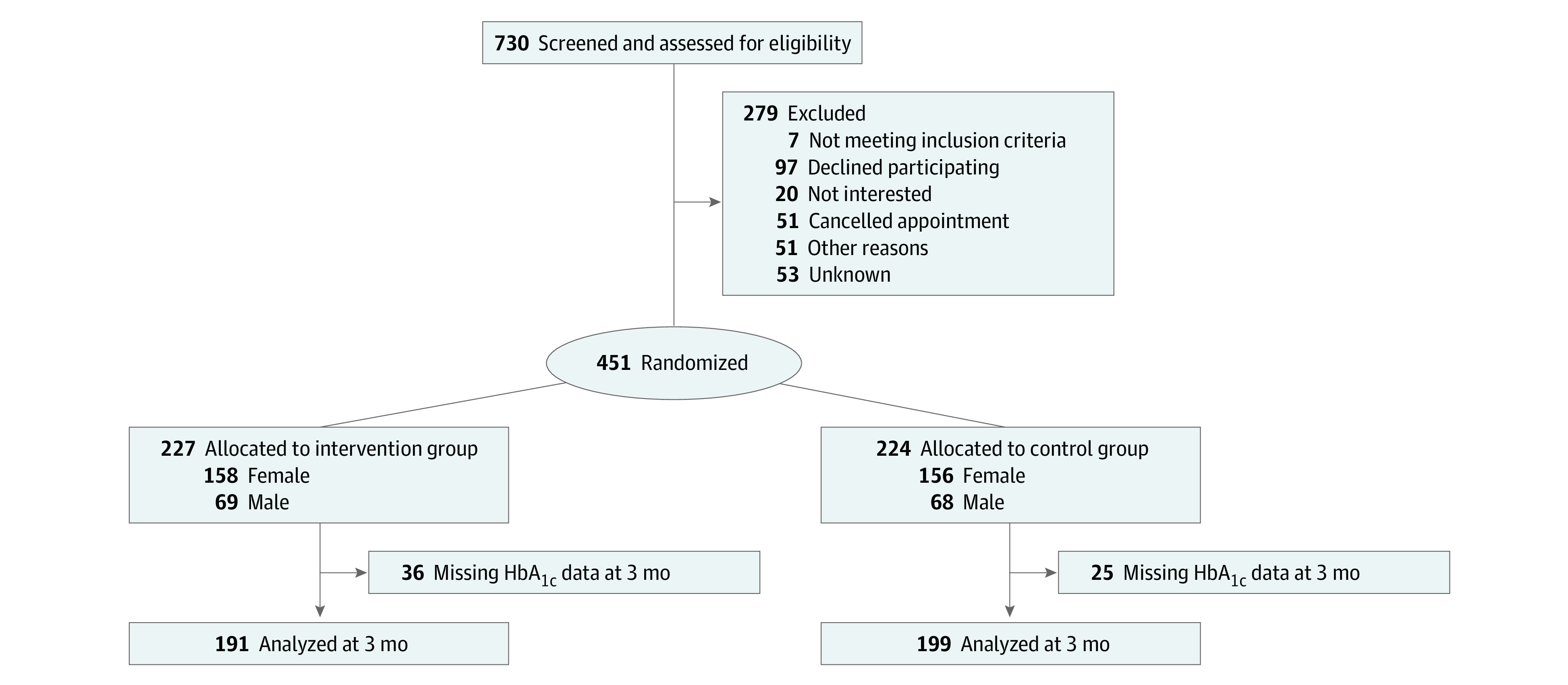
Recruitment, Randomization, and Follow-Up HbA_1c_ indicates hemoglobin A_1c_.

### Intervention Effects

The primary outcome showed improvement in the intervention group compared with the control group in the adjusted model (mean [SD] hemoglobin A_1c_ level, 9.1% [1.7] to 8.4% [1.6] vs 9.4% [1.8] to 8.8% [2]; *P* = .04), but not in the unadjusted model (Table 2). The odds of achieving a hemoglobin A_1c_ less than 8% was 1.5 (95% CI, 1.0-2.4) times higher in the intervention group than the control group, but this difference did not achieve statistical significance (*P* = .06) (eFigure 2 in Supplement 1). There were no significant observed differences in secondary outcomes (Table 2).

**Table 2.** Unadjusted and Covariance-Adjusted Change in Primary and Secondary Outcomes From Baseline to 3 Months

Outcome	Estimate (95% CI)	*P* value^a^	
		Unadjusted	Adjusted^b^
Hemoglobin A_1c_, %			
Intervention	−0.70 (−0.93 to −0.46)	.24	.04
Control	−0.64 (−0.86 to −0.42)		
Systolic blood pressure, mm Hg			
Intervention	0.91 (−1.37 to 3.20)	.31	.72
Control	−2.21 (−4.72 to 0.30)		
Diastolic blood pressure, mm Hg			
Intervention	−0.59 (−1.86 to 0.69)	.54	.14
Control	−0.39 (−1.68 to 0.90)		
Body mass index^c^			
Intervention	−0.24 (−0.76 to 0.28)	.52	.55
Control	0.10 (−0.24 to 0.45)		
Weight, kg			
Intervention	−0.75 (−2.17 to 0.68)	.51	.57
Control	0.19 (−0.55 to 0.93)		
LDL cholesterol, mg/dL			
Intervention	−5.25 (−10.94 to 0.44)	.26	.19
Control	−1.64 (−6.72 to 3.44)		

Abbreviation: LDL, low-density lipoprotein.

SI conversion factors: To convert hemoglobin A_1c_ to proportion of total hemoglobin, multiply by 0.01. LDL cholesterol to millimoles per liter, multiply by 0.0259.

^a^ Covariance-adjusted comparison between study groups of continuous measures at follow-up, accounting for baseline hemoglobin A_1c_ level.

^b^ Adjusted for age to gender to education to family income.

^c^ Body mass index was calculated as weight in kilograms divided by height in meters squared.

### Intervention Acceptability and Narrative Quality

Most participants in the intervention group reported that the video was acceptable (221 of 226 participants [97.8%]) and got their attention (220 [97.3%]). Furthermore, 223 participants (98.6%) reported that they were more confident about managing their diabetes than before they watched the video (self-efficacy); 221 participants (97.8%) reported that the video motivated them to change a specific behavior related to diabetes self-management (Table 3).

**Table 3.** Stories for Change: Diabetes Intervention Acceptability and Narrative Quality

Domain	Participants, No. (%)
What is your general reaction to the intervention?	
Very acceptable	211 (93.4)
Somewhat acceptable	10 (4.4)
Not acceptable	3 (1.3)
Did not answer	2 (0.9)
Does the intervention get your attention	
Yes to very much	213 (94.2)
Somewhat	7 (3.1)
No	3 (1.3)
Did not answer	3 (1.3)
After watching the intervention, rate your confidence about managing diabetes	
Much more confident	196 (86.7)
Somewhat more confident	27 (11.9)
No more confident	2 (0.9)
Did not answer	1 (0.4)
Does watching the intervention make you want to do anything different to manage your diabetes?	
Yes	221 (97.8)
No	5 (2.2)
What do you intend to do differently as a result of watching the intervention?	
Eat a healthier diet	161 (70.6)
Be more physically active	122 (53.5)
Improve blood glucose self-monitoring	47 (20.6)
Take medications as directed	42 (18.4)
Ask others for support	15 (6.6)
Other	8 (3.5)
Narrative quality^a^	
Story identification, mean (SD)	4.8 (0.43)
Story transportation, mean (SD)	4.9 (0.34)

^a^ Narrative quality was assessed on a 5-point Likert scale.

Mean responses for the Narrative Quality Assessment Tool subscales were both high (Table 3). Respondents endorsed a mean (SD) story identification subscale score of 4.8 (0.4) of 5 and story transportation subscale score of 4.9 (0.3) of 5, indicating strong agreement, strong identification, and strong emotional engagement in the intervention group. Participants identified with the storytellers and engaged with the story.

## Discussion

In this randomized clinical trial, a digital storytelling intervention for T2D self-management among Hispanic patients was highly acceptable, with high narrative quality, and could be successfully implemented within diverse primary care clinical settings. The intervention resulted in a modest improvement of glycemic control at 3 months in the adjusted model, but not the unadjusted model. The intervention was developed by a CBPR partnership and represents an example of community informing clinical practice.

To our knowledge, this is the first digital storytelling intervention reported for T2D management. Portions of the logic model were supported by study findings, and others were not. The hypothesis that story identification and transportation would be high among viewers was confirmed. The next hypothesis was that story identification and transportation would result in improved T2D-related self-efficacy and behaviors. Participants in the intervention group reported more confidence (self-efficacy) and motivation to manage their T2D as a result of receiving the intervention, but this did not translate to statistically significant improvements of behaviors. This study builds on work from a narrative-based video intervention among 598 patients with T2D in Australia,^14^ which found improvements in self-efficacy and behaviors at 4 weeks (glycemic control was not assessed). The reasons for this discrepancy may be secondary to the moderate to high hemoglobin A_1c_ values at baseline. Likewise, the lack of change in secondary outcomes of blood pressure and LDL cholesterol may be because mean baseline values were in the reference range. In contrast, suboptimal glycemic control (hemoglobin A_1c_ ≥8%) was a requirement for study eligibility.

This intervention may be clinically relevant due to its high scalability. It had high narrative quality, and participants felt more confident about managing their T2D and felt activated to make specific health-related changes as a result of viewing the intervention, all while achieving a potentially modest improvement on hemoglobin A_1c_ levels. While this brief intervention at a single point in time did not have a robust effect on glycemic control, its characteristics and outcomes suggest that it may be most impactful when combined with a longitudinal, interactive intervention that is culturally and linguistically tailored to Hispanic communities. A previous mHealth intervention tailored to Hispanic patients^35^ showed improvements in glycemic control through an interactive text message platform. Future research should test the combination of the digital storytelling intervention (for upfront activation) with this or other longitudinal mHealth interventions^36^ for synergistic effects. The advantage of these approaches as a supplement to usual care is that they ensure theory and community-informed, culturally tailored messaging alongside longitudinal monitoring. The digital storytelling intervention is freely available through a web-based link and/or software application. This scalability and portability make it an attractive intervention component for a population that is disproportionately affected by T2D, in part, due to lower access to health care. Future research should also determine the sociodemographic and T2D-related factors most likely to predict a favorable response to the intervention so that it may be targeted to patients most likely to benefit.

The intervention was tested in primary care clinical settings. The interventionist was a study staff member, which could translate to medical assistants, community health workers, or nurses during the check-in or rooming process. The intervention could also be used by diabetes educators with individual patients or in group T2D education sessions. We previously conducted a qualitative study that demonstrated the feasibility of using this intervention in community-based group settings through facilitated discussion.^37^ Finally, the intervention could be fully automated at scale via patient portal messages to Hispanic patients with poorly controlled T2D. These different implementation conditions warrant further study.

### Limitations

This study has limitations. Data collection occurred during the COVID-19 pandemic, and associated stressors may have made diabetes self-management challenging. Despite the intentional recruitment of diverse storytellers and participants from distinct sites, it was not possible to reflect the vast heterogeneity of Hispanic populations in the United States. The discussion of diabetes by the culturally congruent study staff members as part of measurements may have contributed to an unanticipated therapeutic effect within the control group. Baseline hemoglobin A_1c_ was higher in the control group than the intervention group (9.4% vs 9.1%). Since larger improvements in hemoglobin A_1c_ are expected for patients with higher baseline values under usual care conditions,^38^ intervention effects may have been blunted. There were small amounts of missing data, and various multiple imputation methods resulted in *P* values for the primary adjusted analysis ranging from *P* = .04 to *P* = .15, which further conditions any conclusion about the impact of the intervention on glycemic control. Additionally, this study is limited by a relatively short-term follow-up period for a chronic disease.

## Conclusions

In this randomized clinical trial, the digital storytelling intervention was highly acceptable and feasibly implemented within primary care settings and resulted in a potentially modest improvement in glycemic control. This was a scalable and portable intervention that may be integrated into clinical and public health practice as part of a longitudinal self-management program for Hispanic adults with T2D.

## Notes

Supplement 1. **eFigure 1.** Logic Model for the Stories for Change Intervention
eMethods.
**eFigure 2.** Covariance-Adjusted Mean Change in Hemoglobin A_1c_ and the Proportion of Participants Who Achieved Hemoglobin A_1c_ Levels of Less Than 8% From Baseline to 3 Months

Supplement 2.
Trial Protocol

Supplement 3.
Data Sharing Statement
