# Supplementary material for: Digital Storytelling Intervention for Hemoglobin A1c Control Among Hispanic Adults With Type 2 Diabetes: A Randomized Clinical Trial
Source: JAMA Netw Open. 2024 Aug 2;7(8):e2424781. doi: 10.1001/jamanetworkopen.2024.24781 (PMC11297376; doi:10.1001/jamanetworkopen.2024.24781)
Supplement: Supplement 1. — eFigure 1. Logic Model for the Stories for Change Intervention eMethods. eFigure 2. Covariance-Adjusted Mean Change in Hemoglobin A1c and the Proportion of Participants Who Achieved Hemoglobin A1c Levels of Less Than 8% From Baseline to 3 Months [file jamanetwopen-e2424781-s001.pdf]

Supplemental Online Content

Wieland ML, Vickery KD, Hernandez V, et al. Digital storytelling intervention for hemoglobin A_{1c} control among Hispanic adults with type 2 diabetes: a randomized clinical trial. *JAMA Netw Open.* 2024;7(8):e2424781. doi:10.1001/jamanetworkopen.2024.24781

eFigure 1. Logic Model for the Stories for Change Intervention

eMethods.

eFigure 2. Covariance-Adjusted Mean Change in Hemoglobin A_{1c} and the Proportion of Participants Who Achieved Hemoglobin A_{1c} Levels of Less Than 8% From Baseline to 3 Months

This supplemental material has been provided by the authors to give readers additional information about their work.

© 2024 Wieland ML et al. *JAMA Network Open.*

eFigure 1. Logic Model for the Stories for Change Intervention

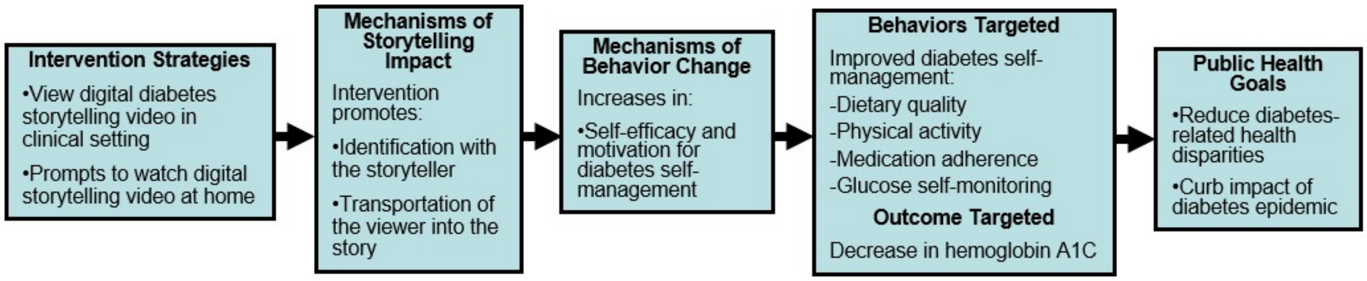

© 2024 Wieland ML et al. *JAMA Network Open.*

eMethods.

Digital Storytelling Intervention for Type 2 Diabetes Self-Management among Hispanic Adults: A Randomized Clinical Trial

Study Setting: Practice Characteristics

- Hennepin Healthcare, Minneapolis, MN. Hennepin Healthcare is a large public academic healthcare institution that provides primary care to approximately 90,000 patients in Hennepin County, MN, a large proportion of whom are Hispanic.
- Mountain Park Health Center (MPHC), Phoenix, AZ. MPHC is a community health center with more than 30 years of experience. MPHC provides primary care for approximately 60,000 patients. Demographically, the vast majority of patients seen at MPHC are Hispanic.

Stories for Change: Diabetes Digital Storytelling Intervention

A web-based link to the Stories for Change: Diabetes digital storytelling intervention can be found here:

Stories for Change (youtube.com)

Scan this QR code for access to the Stories for Change: Diabetes software application:

© 2024 Wieland ML et al. *JAMA Network Open.*

Stories for Change study sample size considerations

Two meta-analyses of diabetes education interventions among Hispanic patients[1,2] and our preliminary data from a non-randomized pilot study[3] suggested that we could expect an effect size between 0.25 and 0.31 standard deviations. Sample sizes to detect these effect sizes, assuming 80% power, a two-tailed alternative and a 5% Type I error rate, were 252 and 145 participants per group, respectively. With 200 participants per group, there was 80% power to detect an effect size of 0.28 standard deviations in the primary outcome. Accounting for approximately 12% loss to follow-up, 455 participants were recruited.

1. Ferguson S, Swan M, Smaldone A. Does diabetes self-management education in conjunction with primary care improve glycemic control in Hispanic patients? A systematic review and meta-analysis. *Diabetes Educ*. Aug 2015;41(4):472-84. doi:10.1177/0145721715584404
2. Ricci-Cabello I, Ruiz-Perez I, Rojas-Garcia A, Pastor G, Rodriguez-Barranco M, Goncalves DC. Characteristics and effectiveness of diabetes self-management educational programs targeted to racial/ethnic minority groups: a systematic review, meta-analysis and meta-regression. *BMC Endocr Disord*. Jul 19 2014;14:60. doi:10.1186/1472-6823-14-60
3. Wieland ML, Njeru JW, Hanza MM, et al. Pilot Feasibility Study of a Digital Storytelling Intervention for Immigrant and Refugee Adults With Diabetes. *Diabetes Educ*. Aug 2017;43(4):349-359. doi:10.1177/0145721717713317

Assessment of missing data

Missing data was relatively rare for the measures included in the final model with household income having the largest number of missing values (8.5%). All other factors in the model had less than 2% missing data, most less than 1%. However, as a further sensitivity analysis, multiple imputation (MI) was conducted for all analyses to account for missing data. MI assumes that any data to be imputed is missing at random (MAR) meaning that missingness found in the data is not the result of the data generation method, in this case survey completion, blood sample, etc. Several MI approaches, including fully conditional specification (FCS), EM algorithm, Markov chain Monte Carlo (MCMC), propensity score methods, and predictive mean matching methods were explored. Each missing value was imputed with a value drawn from the observed distribution which preserves the variance-covariance matrix of the final model. This was done 50 times and the results of the models run on each imputed dataset were then pooled.

© 2024 Wieland ML et al. *JAMA Network Open.*

eFigure 2. Covariance-Adjusted Mean Change in Hemoglobin A_{1c} and the Proportion of Participants Who Achieved Hemoglobin A_{1c} Levels of Less Than 8% From Baseline to 3 Months

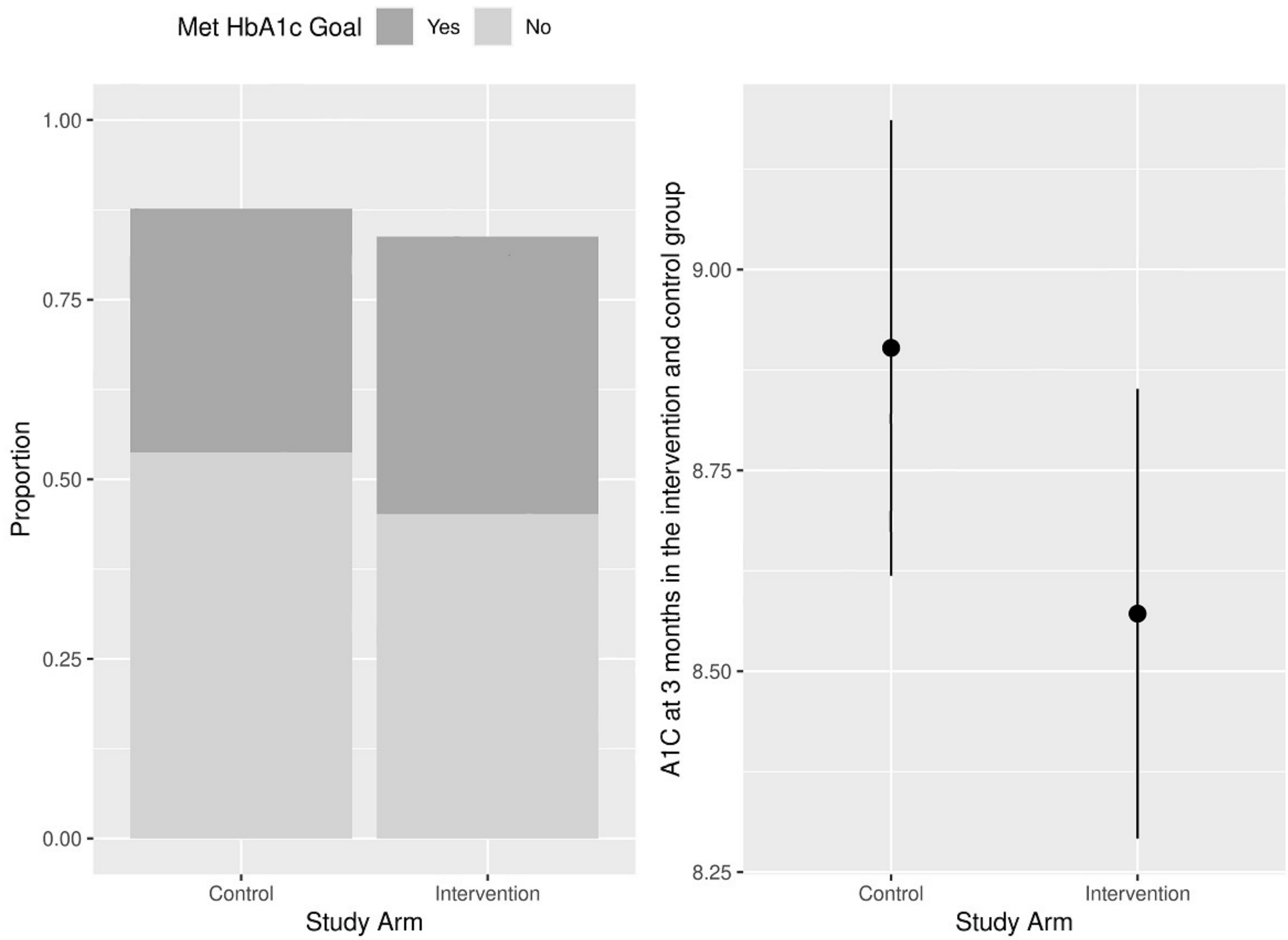

© 2024 Wieland ML et al. *JAMA Network Open.*
