# Supplementary material for: Digital Storytelling Intervention for Hemoglobin A1c Control Among Hispanic Adults With Type 2 Diabetes: A Randomized Clinical Trial
Source: JAMA Netw Open. 2024 Aug 2;7(8):e2424781. doi: 10.1001/jamanetworkopen.2024.24781 (PMC11297376; doi:10.1001/jamanetworkopen.2024.24781)
Supplement: Supplement 2. — Trial Protocol [file jamanetwopen-e2424781-s002.pdf]

WORKING PROTOCOL**

Stories for Change: Digital Storytelling for Diabetes Self-Management among Hispanic Adults

Funding: NIH NIDDK, R01 DK113999-01A1

Study Personnel

Principal Investigators
Mark Wieland, MD, MPH, Mayo Clinic
Irene Sia, M.D., MSc, Mayo Clinic

Co-Investigators
Christi A. Patten, PhD, Mayo Clinic
Jane W. Njeru, MBChB, Mayo Clinic
Jeff A. Sloan, PhD, Mayo Clinic

Other personnel
Paul Novotny, MS, Statistician
Sheila Iteghete, Project Coordinator

Consortium Study Personnel
<u>Hennepin Healthcare, MN</u>
Katherine Vickery, MD, MPH, Site PI/Co-Investigator
Rachel Silva, MD, MPH, Co-Investigator
Becky Ford, PhD, Project Manager
Breidy Garcia, Research Assistant

<u>Mountain Park Health Center, AZ</u>
Davinder Singh, MD, Site PI/Co-Investigator
Valentina Hernandez, Project Manager
Crystal Ramos, Study Coordinator
Sandra Bahena, Clinical Research Coordinator
Elizabeth Hernandez, Research Assistant

**The working protocol is a detailed description of how the funded grant will be implemented.

Specific Aims

Hispanic adults are twice as likely to have type 2 diabetes mellitus (T2D) and 1.5 times more likely to die from the disease than non-Hispanic whites[1,2]. These disparities are mediated, in part, by less healthful levels of physical activity, dietary quality, medication adherence, and self-monitoring of blood glucose than non-Hispanic whites[3-6]. Innovative approaches that arise from affected communities are needed to address these health disparities. Community-based participatory research (CBPR) has been successful in targeting health issues among Hispanic and immigrant populations[7-9]; CBPR is an engaging and effective approach for addressing health behaviors in a sociocultural context[10]. In 2004, our research team developed a CBPR partnership between immigrant communities and academic institutions in Rochester, Minnesota. Since its inception, Rochester Healthy Community Partnership (RHCP) has matured, developed a community-based research infrastructure, and become productive and experienced at deploying data-driven programming and outcomes assessment among diverse immigrant populations in an effort to reduce health disparities[11,12].

Storytelling or narrative-based interventions are designed to incorporate culture-centric health messaging to promote behavior change among vulnerable populations[13,14]. Previous research has shown that narrative based video interventions are more likely to elicit positive motivations for healthful behaviors than non-narrative video messaging for cancer screening[15,16] and smoking cessation[17] among viewers. A single randomized trial has evaluated the impact of a narrative-based video intervention on chronic disease management, showing significant improvement in hypertension outcomes[18]. Digital storytelling interventions are narrative-based videos elicited through a CBPR approach to surface the authentic voices of individuals overcoming obstacles toward engaging in health promoting behaviors[19] to shape positive health behaviors of viewers through influences on attitudes and beliefs[20].

RHCP partners from Hispanic communities identified T2D as a priority area for intervention, and have co-created each of the formative phases leading up to this proposal. Narrative theory and social cognitive theory formed the conceptual basis for intervention development. Our study team conducted surveys[21,22] and focus groups[23] to derive the approach and personnel for building an authentic intervention that was created in a digital storytelling workshop where stories about diabetes self-management were captured, recorded, and edited to derive the final intervention products in video format[23]. The respective digital storytelling videos were pilot tested with 25 patients across healthcare institutions in Minnesota and Arizona. The intervention was rated as highly acceptable, culturally relevant, and perceived as efficacious for motivating behavioral change[24].

The overall objective of this application is therefore to assess the efficacy of a digital storytelling intervention derived through a CBPR approach on self-management of T2D among Hispanic adults.

Specific Aim #1: Evaluate the efficacy of a community-derived digital storytelling intervention among Hispanic adults with poorly controlled T2D.

Hypothesis 1: The digital storytelling intervention will significantly improve glycemic control and diabetes self-management behaviors among Hispanic adults with poorly controlled T2D compared with controls.

We will conduct a two-group randomized controlled trial in primary care clinical settings at two healthcare institutions among 455 Hispanic adults with poorly controlled T2D (hemoglobin A1c≥8%). The intervention group will view the 12-minute digital storytelling video. The comparison group will receive usual clinical care. The primary outcome will be glycemic control as measured by hemoglobin A1c 3 months after intervention delivery. Secondary outcomes will include diabetes self-management behaviors, blood pressure, LDL-cholesterol, and body mass index. The impact of concomitant covariates, including sex, age, and socio-economic status, on the sensitivity of the intervention effect will also be explored.

Specific Aim #2: Explore the effect of the digital storytelling intervention on proposed theory-based mediators of change.

Hypothesis 2: Digital storytelling intervention effects on diabetes outcomes will be mediated by social cognitive constructs (self-efficacy, outcome expectancy, social support) and narrative factor constructs (story identification and transportation).

We will assess whether these theoretical factors impact the relationship between intervention effects on diabetes outcomes. A conceptual framework for potential mechanisms will be constructed to describe how digital storytelling may improve these outcomes.

This work is significant because it tests a scalable, low cost intervention targeting vulnerable populations implemented in primary care settings with the opportunity for rapid translation to practice. The innovation comes from utilizing a digital storytelling intervention created through a CBPR process for T2D management among Hispanic adults. By using rigorous and transparent methods, we will establish a disseminable framework for community participation in digital storytelling production for chronic disease management, providing a model that may be applied to other communities and health priorities.

RESEARCH STRATEGY

1. Significance

Racial and ethnic minorities with type 2 diabetes mellitus (T2D) in the United States (U.S.) are more likely to develop complications and die from the disease than the general population[25]. The Hispanic/Latino population is a heterogeneous mix of people born in and outside the U.S. who make up 17% of the population and who contributed to the majority of U.S. population growth (56%) over the last decade. While there is significant variability between different Hispanic/Latino groups[26], the age-adjusted prevalence of T2D among these populations as a whole was 22.6% in 2012, compared with 11.3% for non-Hispanic whites[1]. Likewise, Hispanic groups are less likely to achieve optimal glycemic control[27] and have 1.5 times higher age-adjusted diabetes-related mortality than non-Hispanic whites[2].

Hispanic groups are less likely to achieve healthy levels of physical activity[3], eat a healthful diet[4], adhere with prescribed medications for T2D[5] or achieve targets for self-monitoring of blood glucose[6] than non-Hispanic whites, each of which are critical domains for the self-management of T2D that can delay complications associated with the disease[28]. These behaviors are influenced, in part, by discrepancies in socioeconomic position, discrimination, access to healthcare, and access to health-promoting neighborhoods and environments[29]. These structural factors interact with psychosocial constructs and health behaviors in complex ways through the life course[30]. Higher levels of acculturation (however defined) among Hispanic groups are generally associated with improvements in some structural barriers to diabetes care (e.g., access to healthcare, education level), but significant worsening of diabetes risk and diabetes-related behaviors (e.g., dietary quality)[31-35]. Likewise, low health literacy is an important distal mediator of diabetes-related health disparities among Hispanic groups[36].

This mechanistic complexity of diabetes-related health disparities highlights the difficulty in designing interventions to promote healthy behaviors among Hispanic and immigrant populations[37,38]. Community-based participatory research (CBPR) is a means to collaboratively investigate healthy living interventions, whereby community members and academics partner in an equitable relationship through all phases of the research.[39,40] This is an approach to research that is well suited to intervention work that addresses the interplay between health behaviors and the social determinants of health, such that it empowers communities, promotes understanding of culturally pertinent issues, and targets the multi-faceted barriers to health.

Narrative-based (storytelling) interventions incorporate culture-centric health messaging to promote behavior change among vulnerable populations[13,14]. Storytelling interventions for behavior change use narratives that resonate with target populations either through direct quotes from representative members or through story compositions inspired by culturally embedded informants[15,41,42]. The ways in which stories elicit behavioral responses among target populations have been conceptualized as *identification* and *transportation*. When narrative is drawn from within populations, identification with the storytellers is an important step for engagement, empowerment, and re-framing social norms of the listener[43-47]. Transportation of the listener into the story is likewise important to generate persuasive effects for behavior change[45,48-51]. For T2D, a group-based storytelling intervention in the United Kingdom demonstrated feasibility and acceptability of this approach among immigrants with T2D in face-to-face (workshop) settings.[52] Indeed, storytelling interventions may be especially powerful among immigrants when communication barriers limit applicability of existing health education opportunities.[53] Furthermore, this approach is promising in populations with a strong oral tradition[54], including those from Latin America[55].

Narrative-based video interventions, where story components are incorporated into health communication media through a variety of ways, provide the opportunity for wide distribution and inclusion of consistent content to promote health behavior change. It has been demonstrated that narrative-based video interventions are more likely to elicit positive motivations for healthful behaviors than non-narrative video messaging for cervical cancer screening[14], breast cancer screening[15,16], and smoking cessation[17] among African American and Hispanic populations. Our review of the literature identified three published studies evaluating the impact of narrative-based video interventions on health behaviors or other outcomes. Larkey and colleagues found that engagement with a storytelling video for colorectal cancer screening facilitated positive attitudes associated with behavior change, but no difference in screening rates when compared with a personal risk tool among low-income patients[56]. In the first randomized trial of a storytelling video intervention for chronic disease management, Houston and colleagues demonstrated efficacy comparable to adding a medication for treatment of uncontrolled hypertension among African American women viewers.[18] Finally, Campbell and colleagues established the promising role of storytelling videos in management of diabetes through a randomized trial among patients with T2D in Australia, which demonstrated significant improvements in self-reported diabetes self-management (diet, exercise, and glucose monitoring) among viewers[57].

<u>Digital storytelling interventions</u> are narrative-based videos elicited through a CBPR approach to surface the authentic voices of participants overcoming obstacles to health promoting behaviors[19]. They differ from other forms of narrative-based videos in that participants are central to the production of knowledge. Through a group-based digital storytelling workshop, storytellers build their own narrative, choose images and sounds that best represent their experiences, and are guided through hands-on computer editing. Through a process of social construction, individuals and groups derive concepts and actions to co-create meaning. This differs from the communication paradigm where "experts" generalize an experience for a community, e.g., documentary story. Instead, participants construct their own experiences in a group setting of peers who, through reaction and feedback, contribute in turn to the shared understanding of the individuals' experiences. This process has been used to empower participants through personal reflection[58-60] and as a tool for health advocacy[61], but the resultant videos can also shape health behaviors of viewers through influences on attitudes and beliefs[20]. Formative studies have demonstrated cultural acceptability and emotional engagement in response to a digital storytelling intervention derived through CBPR processes for cancer education among Alaskan natives[54,62]. We are not aware of any published studies evaluating the impact of digital storytelling interventions on health outcomes of viewers.

With this study, we therefore propose to evaluate the impact of a digital storytelling intervention on health outcomes in Hispanic adults with T2D. Only one of the studies cited above reported sex differences, finding that female sex was associated with positive behavior change after exposure to the narrative video intervention[56]. Therefore, we will explore the potential role of sex as a moderator of the effect of our proposed intervention. This study is significant because it will evaluate a scalable, low cost, high reach intervention targeting vulnerable populations whose health is critical to the future of this country. Furthermore, this intervention will be implemented in primary care settings with the opportunity for rapid translation to practice. Finally, this work will advance the science of participatory intervention development among Hispanic populations while providing a model (digital storytelling) that may be applied to other communities.

<u>2. Innovation</u>

The research proposed in this application is innovative because it focuses on digital storytelling to promote health behavior change in an underserved population. Authors of a recent systematic review identified the need for more culturally tailored chronic disease management interventions that incorporate health technology[63], but there are no published reports of interventions using narrative (storytelling) media for diabetes among minority or immigrant populations. Moreover, community participation is a particularly powerful mechanism to maximize cultural and linguistic acceptability of the intervention, adding to the innovation. Furthermore, we have built a theoretical perspective within the context of the study design, extending beyond our pilot study assessment of social cognitive constructs to include storytelling factor measures to contribute knowledge on the psychosocial factors associated with behavior change. Finally, by using rigorous and transparent methods, we will establish a disseminable framework for community participation in digital storytelling production for chronic disease management, providing a model that may be applied to other communities and health priorities.

3. Approach

This work builds on our thirteen years of experience with CBPR through Rochester Healthy Community Partnership (RHCP) http://rochesterhealthy.org/. Past experience with CBPR interventions and partnership development has led us to collectively consider and derive an intervention to improve T2D management among Hispanic adults in our community through a CBPR approach using methods of digital storytelling.

CBPR partnership experience and narrative

In 2004, a community-academic partnership developed between Mayo Clinic and Hawthorne Education Center (HEC), an adult education center that serves approximately 2,500 immigrants per year. This partnership has matured by formalizing operating norms, adopting CBPR principles, adding many dedicated partners, conducting community health assessments, and completing several CBPR projects. The mission of RHCP is to promote health and well-being among the Rochester community through CBPR, education, and civic engagement to achieve health equity[64]. The definition of community by RHCP has been delineated by individual ethnic/cultural groups (e.g., Hispanic/Latino) as well as a shared sense of experience of recent immigration, low English language and health literacy, and relatively low socioeconomic position among individuals from these heterogeneous groups. Moreover, the partnership includes community-based organizations that work with members of these communities. RHCP has developed an effective **community-based research infrastructure** that has facilitated human subjects protection training for community partners[65]; more than 30 community partners have completed our training in qualitative moderation and analysis[66].

RHCP has become **productive and experienced** at deploying data-driven programming and outcomes assessment among immigrant and populations[11,12]. RHCP obtained extramural funding in 2008 through the NIH Partners in Research Program (R03 AI 82703) to strengthen the CBPR partnership through development of a culturally sensitive educational and health literacy infrastructure for immigrant populations. In 2011, RHCP received funding through the NIH Program Announcement for Community Participation in Research (R01 HL 111407) to conduct a randomized controlled trial of a family-based behavioral lifestyle intervention. Community and academic partners have conducted every phase of research and programming together, and disseminate research results jointly at community forums and academic meetings.

<u>Experience with conducting a randomized controlled trial among Hispanic populations.</u> RHCP community and academic partners recently co-created and tested a multi-component physical activity and nutrition intervention with Hispanic and Somali families through a CBPR approach. Target enrollment was achieved (40 families; 151 individuals) and randomization was successful[12]. All participating families assigned to the intervention group received 100% of the intervention with very high fidelity of the intervention[67]. At 12 months, study retention was high (91% of families, 82% of individuals). We found statistically significant improvements in dietary quality among participants in the intervention group compared with wait list controls[68].

<u>Rationale for addressing T2D.</u> Previous community needs assessments including an open-ended survey among 175 immigrant adults at one of the community partner sites (HEC) and a survey of RHCP community leaders in 2010 revealed that T2D was among the top health priorities for Hispanic populations in our city. Their other top concerns (physical activity, nutrition, obesity, hypertension) mirror national trends reflecting increasing obesity and associated co-morbidities among immigrants after arrival to the US[69]. RHCP members reflected on the seemingly high impact of T2D on their communities and the overwhelming burden this disease leaves on individuals and their families as they strive and struggle to understand and manage the condition.

<u>Rationale for digital storytelling.</u> During RHCP brainstorming sessions, community partners were clear that the current systems for T2D management among Hispanic adults do not address the social, linguistic, or cultural needs of their communities, and that these limitations negatively impact diabetes-related outcomes. Further, low health literacy was implicated as a common pathway for sub-optimal T2D care. The idea of storytelling arose from this paradigm as a means to mitigate these variables. Indeed, there are data to suggest that storytelling is a promising approach for interventions to improve T2D management among immigrants[52]. Finally, RHCP's experience with the community-based qualitative work needed to build a foundation for storytelling[70-73], and RHCP's past experience with digital video production for health promotion among immigrant groups[74] were seen as predictors of successful implementation of a digital storytelling intervention.

<u>Rationale for focusing on Hispanic populations.</u> RHCP has a history of successful collaborative work between many different immigrant groups (and non-immigrant Latinos) towards common goals. Our recent RHCP

partnership evaluation highlighted the positive impact of this unique collaboration on community building, collective advocacy, and health outcomes[75]. For this project, community leaders and community-based organizations from both Hispanic and Somali populations have taken leadership roles for intervention co-creation. Work with these two groups occurred both in parallel and in collaboration, such that best practices and learning opportunities have been shared throughout the process. Likewise, RHCP academic partners have documented local diabetes-related disparities for both groups[76-78]. For this grant application, community and clinical partners elected to focus on the Hispanic population because of the disproportionately large number of eligible patients within this group, and to reduce implementation complexity of the research procedures. Because of the relatively low number of eligible Somali patients, we intend to test the intervention in this group on a smaller scale and through a different funding mechanism.

In summary, over the last 13 years, we have formalized a successful CBPR partnership, and matured into a broad network of dedicated partners uniquely suited to conduct the proposed study.

Preliminary data

RHCP meeting minutes. Based on a shared desire to address T2D, brainstorming sessions were conducted at monthly RHCP meetings. These sessions drew on past partnership and personal experiences, and lessons learned to inform an intervention with the highest likelihood for success. Digital storytelling was embraced as a framework. Building on effective components of past strategies, a mixed community-academic RHCP grant writing sub-committee was established to prepare this grant proposal. Community leaders inside and outside of the Alliance for Chicanos, Hispanics, and Latin Americans co-created this work.

Step 1: Diabetes survey. Drawing from existing validated instruments, a diabetes survey was created by RHCP to assess diabetes attitudes, knowledge, and behaviors among community members with T2D. The survey was adapted and pilot-tested to be culturally and linguistically appropriate through an RHCP-derived process for survey development and translation through a CBPR approach[21]. Survey administration was performed by community partners among 78 participants (39 Hispanic, 39 Somali) with T2D. Respondents reported several barriers to optimal diabetes management for physical activity and glucose self-monitoring, as well as a high burden of disease and negative perceptions of diabetes. High participant engagement in disease management, self-efficacy, and social support were important assets[22].

Step 2: Diabetes focus groups. We conducted three focus groups among a subset of Hispanic survey participants for a dual purpose: 1) to further inform the intervention as it related to four domains of diabetes self-management (physical activity, healthful diet, medication management, self-monitoring of blood glucose); and, 2) to identify persuasive storytellers for the intervention development. Groups were led by language-congruent RHCP community partners trained in focus groups moderation[66]; the qualitative results achieved our study objectives[23]. Storytellers were selected by community partners on the basis of these focus groups data and consensus about who they wanted to represent them as having compelling stories.

Step 3: Digital storytelling intervention development: Four gifted storytellers identified in the focus groups were recruited by RHCP community partners to participate in the story building process. In February 2015, these participants completed a 4.5-day story building workshop with RHCP staff and two facilitators from the Center for Digital Storytelling (CDS, www.storycenter.org). CDS has more than 20 years of experience surfacing authentic voices around the world through participatory media creation. Through their Silence Speaks Initiative (www.silencespeaks.org), CDS has experience coordinating complex projects in public health that involve multiple stakeholders, working cross-culturally in multiple languages.

At the end of the workshop, all participants for this project had created powerful stories about their struggles and accomplishments related to four domains of diabetes self-management: physical activity, dietary quality, medication adherence, and self-monitoring of blood glucose. Stories reflected on motivation and self-efficacy for management of these components. An important aspect of this method of digital story production is that all steps, from the initial consideration of what story to tell, scripting/storyboarding, visuals, music, compiling and editing are in the hands of the storytellers. Minor post-production editing was performed by CDS and RHCP staff/community partners, including the addition of a brief introduction and concluding educational message for the Spanish language videos[23]. Participants provided written consent for dissemination of the final products.

Step 4: Pilot testing of the intervention: The 12-minute digital intervention package was viewed individually by 25 participants (52% female) with T2D in clinical settings at the institutions participating in the work described in this proposal (Hennepin County Medical Center, Minneapolis, MN and Mountain Park Health Center, Phoenix, AZ), as well as at clinical sites in Rochester, MN. Eligible patients were identified through existing registries of primary care patients with T2D at each institution. Recruitment and enrollment was conducted among sequential eligible patients before or after clinic visits at each site. There were no screening failures (i.e., all patients identified as eligible in diabetes registries met inclusion criteria), and only one patient approached for the study declined participation. The intervention was delivered in the clinical setting following a regularly scheduled visit to the patient's primary care clinic. Recruitment, enrollment, and intervention delivery was completed within one week at two institutions, and within four weeks at the other institutions.

After participating in the intervention, a cross-sectional face-to-face structured survey instrument, delivered by a language-congruent study staff, was utilized to assess intervention acceptability and motivational constructs consistent with our theoretical framework. The evaluation assessed the degree to which the intervention motivated the patient to improve self-management of diabetes overall and across the four behavioral domains. The evaluation also assessed overall acceptability of the intervention and cultural relevance. Items for this survey were adapted from a health communication assessment tool produced by the National Cancer Institute[90]. Results demonstrated high acceptability and cultural relevance, with 100% of participants reporting that the intervention got their attention, was interesting, and was useful. In an open-ended question, participants reported a range of "main messages" coinciding with each of the behavior domains[24].

- 25 (100%) participants reported that the video motivated them to change a specific behavior related to diabetes self-management.
- 24 (95%) participants reported that they were more confident about managing their diabetes (self-efficacy) than before they watched the video.
- 25 (100%) of participants reported that they would show it to family members or friends with diabetes if made available.

We obtained follow-up clinical data from the electronic medical records for the subset of patients that viewed the intervention who were Hispanic/Latino and had a baseline hemoglobin A1c>7% (n=13).

- There was a mean reduction in hemoglobin A1c of 1.5% from baseline to the first follow-up value at *three to six months* (p=0.03)[24].

We then derived a control cohort of Hispanic patients (n=13) matched to each intervention patient by site and baseline hemoglobin A1c (+/- 0.5%). We abstracted follow-up blood test results up to *six months* after baseline for all patients. Patients in the intervention group had a hemoglobin A1c that was 37% times the standard deviation lower than the control group, a moderate effect size (Table 1)[24]. Hence, the estimated effect size gleaned from this small, non-randomized pilot study suggests the possibility of a clinically relevant improvement in T2D outcomes as a result of intervention exposure.

Table 1. Pilot study of the digital storytelling intervention among Hispanic adults with poorly controlled T2D

	Intervention Group (n=13)	Control Group (n=13)	p-value
Age, mean (SD)	54.0 (12.7)	56.8 (7.7)	0.53
Female sex, n	6	6	1.00
Baseline hemoglobin A1c, mean % (SD)	10.3 (2.5)	9.9 (2.1)	0.84
Change from baseline to first follow-up A1c, mean(SD)	-1.5 (2.0)	-0.8 (1.8)	0.26

In conclusion, RHCP has spent the last three years developing and evaluating a digital storytelling intervention for T2D self-management among Hispanic adults through a CBPR approach. We have engaged other clinical sites to participate in this project, and we have shown through pilot testing across these institutions that recruitment is feasible, that the storytelling video is acceptable, and that our study team has effective processes for site-specific recruitment and intervention delivery in primary care settings.

Research Design

Overview of the general approach. Our approach has followed established methods for behavioral therapy development research[91,92]. The aim of this research is to evaluate the impact of a digital storytelling intervention

derived through a CBPR approach on T2D outcomes among Hispanic adults with poorly controlled T2D in primary care settings through a randomized clinical trial.

Theoretical approach. The theoretical framework for this work is the application of Social Cognitive Theory (SCT)[93] to Narrative Theory.[13] SCT recognizes the interplay of individual factors, such as self-efficacy to become physically active and social environmental factors like social support, on health behavior. Other key constructs of SCT are outcome expectations or the consequences that result from improved dietary quality (e.g., improved glycemic control). The SCT constructs of self-efficacy, social support, and outcome expectancy have been used successfully to explain self-cares for patients and populations with T2D.[94] Furthermore, self-efficacy and social support are associated with diabetes self-management behaviors among Hispanic populations[95-97]. Narrative Theory recognizes the critical role for narrative (stories) as strategies for navigating experience. In this case, the digital narrative will be used for behavior modeling to facilitate observational learning. Narratives incorporate information, communication and persuasion to encourage desired behavior change.[13] The ways in which the narratives elicit behavioral responses among target populations have been conceptualized as identification with the storytellers[43-47] and transportation of the listener into the story[41-44] as described above.

Our clinical trial will assess the potential effect of the proposed intervention on T2D outcomes (physiologic, behavioral, psychological); it will also identify the potential mechanisms of behavior change (i.e., how the intervention works). Figure 1 provides a logic model framework with intervention strategies and outcomes that are consistent with the theoretical framework.

Figure 1. Logic model of intervention strategies and outcomes

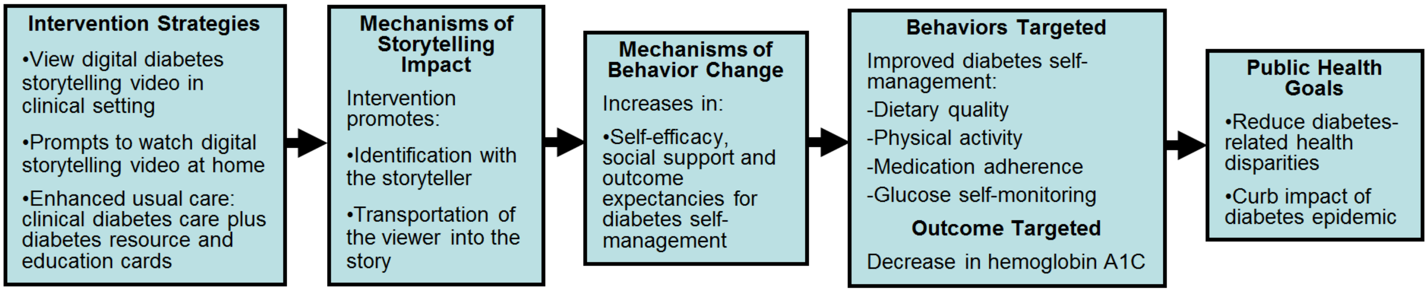

Design Overview

This study (Figure 2) will evaluate the intervention through a two-group, parallel randomized controlled trial in primary care clinical settings across two healthcare institutions among 455 Hispanic adults with poorly controlled T2D (hemoglobin A1c≥8%). The intervention group will view the 12-minute digital storytelling intervention that has been previously pilot-tested, in addition to usual clinical care. The comparison group will receive usual clinical care. The primary outcome will be glycemic control as measured by hemoglobin A1c. Secondary outcomes will include diabetes self-management behaviors, blood pressure, LDL-cholesterol, body mass index, and diabetes process measures. We will also measure covariates of treatment effect consistent with our theoretical framework.

Figure 2. Study Design and Intervention Schedule

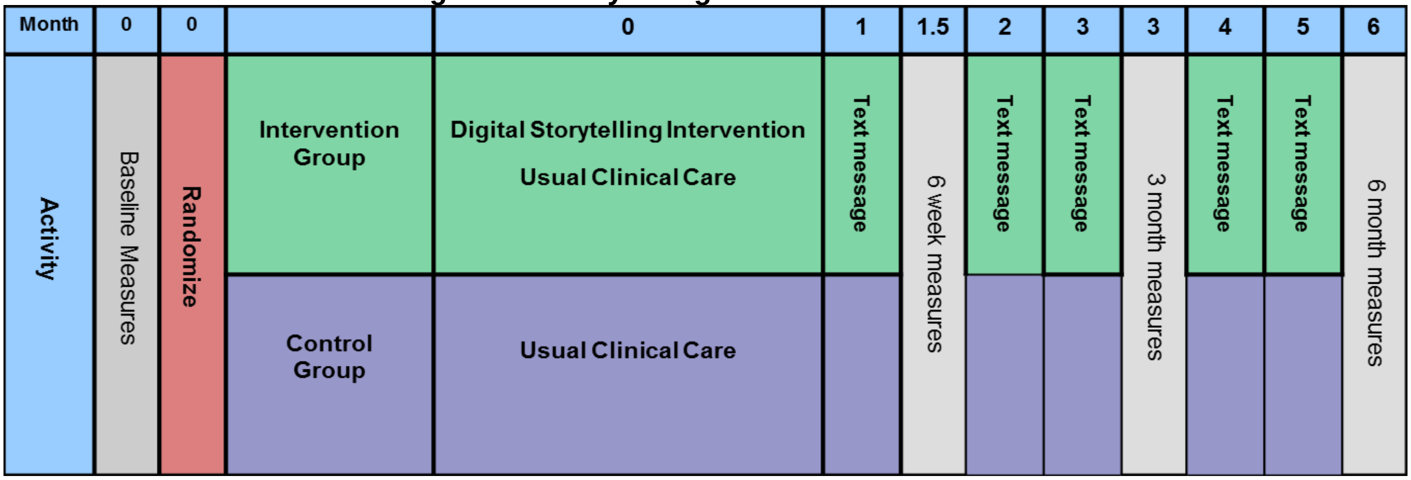

Study Sites

Both clinical sites participated in the pilot testing of the intervention and in refinement of study procedures for the proposed trial. Our letters of support indicate strong investment and commitment to accomplish this study, and study personnel have been identified at both sites:

- Hennepin Healthcare, Minneapolis, MN. Hennepin Healthcare is a large public academic healthcare institution that provides primary care to approximately 90,000 patients in Hennepin County, MN, a large proportion of whom are Hispanic. Members of our study team have worked with HCMC investigators for the past three years on diabetes care for immigrant patients.
- Mountain Park Health Center (MPHC), Phoenix, AZ. MPHC is a community health center with more than 30 years of experience. MPHC provides primary care for approximately 60,000 patients. Demographically, the vast majority of patients seen at MPHC are Hispanic. MPHC has a long history of mission-driven research collaboration with Mayo Clinic.

Rationale for study site selection. The study will take place at Hennepin Healthcare sites and MPHC because of their high volume of adult Hispanic patients with T2D, their strong history of research collaboration with Mayo Clinic, and because of a personal history of collaboration between the PIs and site-PIs (including pilot work for this application). The intervention was initially created with RHCP community partners and Rochester, MN was included as a pilot site. As in the pilot study, Mayo Clinic team members who have substantial expertise with clinical trial coordination and oversight will continue to perform this role (i.e., coordination and oversight) in the proposed study as described below.

Study coordination and oversight. The Mayo Clinic PIs and project coordinator will provide central oversight for the project, including implementation of communication plans with both sites, data handling, data safety monitoring, and data analysis. Mayo Clinic has significant experience and infrastructure as a coordinating center for multi-site trials, including the pilot phase of this study. Study staff from both sites will conduct study procedures under direction of the site PIs (Drs. Vickery and Singh).

We will continue bi-weekly conference calls between Mayo Clinic and study teams at Hennepin Healthcare and MPHC that are on-going in the context of the pilot work and other collaborations. If this proposal is funded, the PIs and project coordinator will meet with the Hennepin Healthcare and MPHC study teams separately for a kickoff retreat followed by quarterly face-to-face meetings.

Study Participants

Eligibility criteria will include 1) self-identify as Hispanic or Latino, 2) age 18 to 70 years, 3) receive primary care at one of the participating clinical sites, 4) at least one office visit within the previous 12 months to the primary care site, 5) diagnosis of T2D in the medical record, 6) T2D diagnosis for six months or longer, 7) most recent hemoglobin A1c≥8%, and 8) intention to continue to receive medical care at the recruitment clinic for the next six months. The value of 8% is higher than the goals established by the American Diabetes Association Guidelines (hemoglobin A1c<7%)[98], but lower than the definition of poorly controlled T2D established by the Center for Medicare & Medicaid Services Core Quality Measures Collaborative (hemoglobin A1c>9%)[99]. It is common practice to increase an individual patient's goal hemoglobin A1c value in the context of clinical and social co-morbidities, but this goal value rarely exceeds 8%. Furthermore, the Minnesota Department of Health

has defined poorly controlled T2D as hemoglobin A1c≥8% for the purposes of mandatory reporting by clinical sites across the state[100]. Therefore, we will use this value in keeping with common clinical practice to determine eligibility for the study.

The first step in screening for eligible participants is identification of Hispanic patients at both study sites with T2D. Per state-wide reporting mandates, each site maintains registries of their primary care patients with diabetes and the most recent hemoglobin A1c value for each patient. The second step in screening is to identify Hispanic adults within the subset of patients with the most recent hemoglobin A1c≥8% at both sites. Both sites have identified mechanisms for accurately identifying eligible patients (see letters of support). *In our pilot study, we identified 3,025 eligible patients across both sites.* Based on our pilot data, we expect to recruit approximately 50% women. Children were not included because of perceived community need that resulted in an intervention created for adults, and because of the relatively higher prevalence of T2D among adults.

Recruitment, Enrollment, Randomization, and Retention

Once eligible patients are identified from institutional registries, the next office appointment for any diabetes-related indication (e.g., chronic disease management provider visit, diabetes educator visit, dietician visit, diabetes group class, etc.) will be recorded for each patient. These patients will be contacted by telephone by a language-congruent study staff member to introduce them to the study and to assess preliminary interest in participation. Patients will be sequentially recruited at their next office visit until the target accrual is reached. Patients may also be recruited, consented and participate in study activities over the phone.

A language-congruent study staff member will confirm eligibility criteria with the patient and obtain written informed consent in an exam or private room before or immediately after their office appointment. Study staff will then obtain a baseline survey and biometric assessment (see measures) followed by randomization procedures (stratified by gender) and delivery of the digital storytelling intervention (for the intervention group). Each site has developed slightly different protocols for effective recruitment and intervention implementation based on lessons learned from the pilot study. Based on data from our pilot, we expect recruitment and enrollment to occur over a period of no more than 24 months across all sites with the following distribution:

- Hennepin Healthcare: N=240 participants enrolled.
    - Medicine Clinic
    - East Lake Clinic
    - Richfield Clinic
- Mountain Park Health Center: N=215 participants enrolled.

Participant retention will be facilitated in five ways. First, potential participants will confirm the eligibility criteria stating that they expect to receive medical care at the recruitment clinic for at least the next six months (above). Second, appointment cards for both subsequent study visits will be provided to participants after they have completed enrollment. Third, language-congruent study staff will contact patients by phone to remind them about upcoming study visits. Fourth, participants who do not return phone calls from study staff will be sent a letter asking them to call the study coordination to reschedule the appointment. Fifth, participants will receive remuneration at enrollment and at the of the 3 month visit.

Interventions

Digital storytelling intervention. The digital intervention package, which is 12 minutes long, includes a brief introduction by an RHCP community partner, four stories with transitions, and a short closing educational message re-enforcing the four diabetes self-management behavioral goals (healthful diet, physical activity, medication adherence, glucose self-monitoring). The storytellers include two women and two men; they reflect some of the heterogeneity among Hispanic/Latino subgroups (two Mexican, one Central American, and one South American) in the U.S. After obtaining consent in the clinical setting or over the phone, study staff will deliver the intervention in the exam room,a separate private room or provide patient with a web address to view the video on YouTube from home.

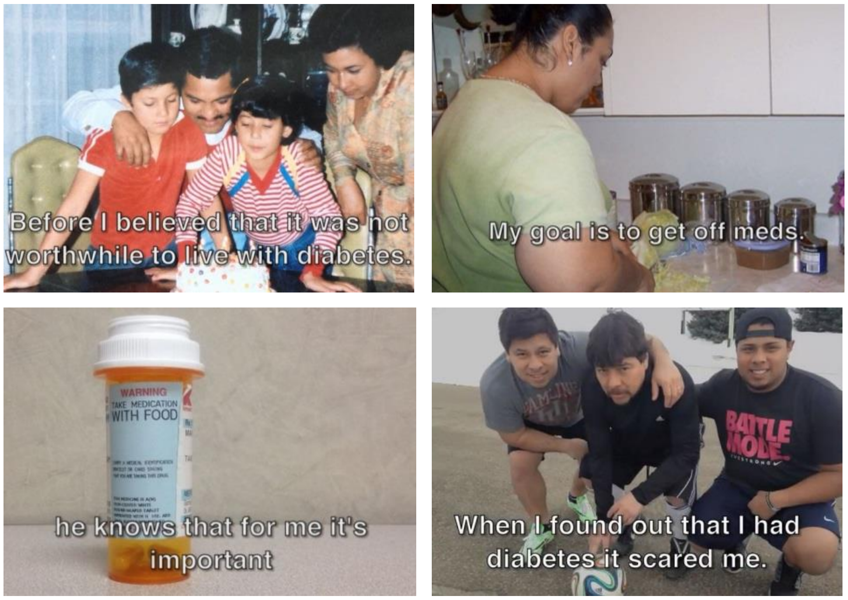

Figure 3. Still images from the existing digital storytelling intervention

Following the video exposure, study staff will ask these questions to each participant and record their answers: 1) What is your reaction to the video?, 2) What was the main message of the video, and 3) Does the video motivate you to make any changes to the way you manage your diabetes (probe positive responses)? These questions will confirm participant receipt of the intervention, which is an important fidelity construct to ensure that participants received and understood the intervention[101].

Participants will be offered a copy of the video as a portable storage device (i.e., “flash drive”) for viewing on a computer, a DVD for viewing on a television, or a web address to watch the video on YouTube. Participants will also be provided access to the storytelling intervention as an application on their mobile phones and/or tablets. In our pilot of the storytelling intervention, all participants cited the use of mobile smart phones. Study staff will offer to assist participants in downloading this application during the baseline visit, and encourage participants to watch the video with family members or friends within their support network.

Rationale for implementation in the clinical setting. The work proposed in this application is intentionally placed on the boundary of the efficacy-to-effectiveness transition[102] in clinical trials. We considered conducting a more traditional efficacy study, where participants would be invited to separate study visits for intervention delivery and measurements. However, interventions that target improvements in diabetes self-management behaviors cannot be easily disarticulated from the clinical setting. The hypothesized patient activation that may occur as a result of exposure to the digital storytelling intervention requires interaction with their healthcare home in order to translate that activation into improved outcomes. Therefore, we pilot tested the intervention in primary care-based clinical settings. We demonstrated feasibility of this approach while adapting intervention implementation for both sites.

Mobile short message service. Mobile short message service, i.e., text messaging, has been shown to have some impact on diabetes self-care[104] and cardiovascular-related lifestyle[105] behaviors. Furthermore, a Cochrane review suggested moderate evidence for increasing self-efficacy for diabetes self-management using mobile phone messaging[106]. Therefore, to increase the likelihood that participants will watch the storytelling video several times during the intervention interval, participants will receive a monthly automated text message (five total) that asks them to self-rate their motivational level and self-efficacy for managing their T2D (0 = no motivation/self-confidence to 10 = extremely motivated/confident), and will recommend that they watch the storytelling intervention if they score lower than a 7. The rationale for the addition of mobile messages is that this intervention augmentation re-enforces concepts consistent with our theoretical framework. It increases

the potential treatment dose while maintaining an intervention package that is highly scalable. As stated above, our pilot data indicate ubiquitous smart phone use among potential study participants.

Control Condition

The control group will participate in the same usual clinical care for diabetes (e.g., visits with primary care physician, nurse, diabetes educator, dietician, etc.) as the intervention group. We will control for study coordinator attention time throughout the enrollment and measurement activities. The control group will be offered the digital storytelling intervention package after the study is completed.

Measures

Participant data will be collected at baseline, six weeks, three months, and six months in their preferred language. Biometric measurements will be obtained during clinic visits at baseline and 3 months. The 3 month visit can occur up to 2 weeks prior to the visit due date and up to 4 weeks after the visit due date, to accommodate scheduling limitations during the COVID-19 outbreak. Participants will complete a survey (administered by same language-congruent study staff) to obtain secondary outcomes and theory-based measures (Table 2) at clinic visits or by phone within 2 weeks following the clinic appointment, and during phone calls at 6 weeks and 6 months. Surveys will be verbally administered to participants by bilingual research staff, and responses will be entered directly into an electronic database. Surveys are not printed and given directly to the participants to complete on their own. Participants will also receive appointment reminders (three total) prior to visits or calls.

Additional clinical data will be collected from the electronic medical records. Investigators and data analysts will remain blinded to group assignment throughout the study.

Demographic, health status, acculturation, health literacy, and diabetes knowledge measures. At baseline, study participants will report on the following demographic data: sex, age, ethnicity, country of birth, annual household income, education level, employment status and housing security. Individuals will report on the following proxies for acculturation: time lived in the U.S., primary language spoken at home, and level of English language proficiency on a 5-point Likert scale[107]. Health literacy will be measured with the Short Assessment of Health Literacy—Spanish and English[108]. Diabetes knowledge will be measured using the Diabetes Knowledge Questionnaire, available in Spanish and English[109].

The following data will be obtained at baseline from manual abstraction of the electronic medical records: date of diabetes diagnosis, diabetes medications, diabetes microvascular complications (nephropathy, retinopathy, neuropathy), and presence or absence of provider-assigned diagnoses of coronary artery disease, cerebrovascular disease, peripheral arterial disease, and hypertension.

Primary outcome measure. The primary outcome measure will be glycemic control as measured by hemoglobin A1c. The rationale for use of hemoglobin A1c as an indicator of diabetes control is based on national and regional data that demonstrate significant disparities in reaching hemoglobin A1c targets for Hispanic populations compared with non-Hispanic whites[27,110]. The importance of glycemic control as part of the comprehensive management of diabetes is well documented, and hemoglobin A1c testing is a well-established strategy to monitor glycemic control in patients with diabetes[111]. Hemoglobin A1c will be measured from whole blood samples obtained and analyzed in a blinded fashion by the clinical laboratories at Hennepin Healthcare and MPHC; both laboratories use methods certified by the National Glycohemoglobin Standardization Program. Both laboratories are certified by the College of American Pathologists.

- Baseline hemoglobin A1c values will be drawn at the baseline study visit, abstracted from the electronic medical records, or reported from an self-check A1c test kit. A1c values drawn up to two weeks prior to the baseline visit date as part of usual clinical care may be used as the baseline A1c value.

- Hemoglobin A1c values at 3 months (primary outcome) will be drawn at the 3 month study visit, or abstracted from the electronic medical records, or reported from an self-check A1c test kit. A1c values drawn up to two weeks prior or 4 weeks after the 3 month visit date as part of usual clinical care may be used as the baseline A1c value.

- Hemoglobin A1c values at 6 months will be abstracted from the electronic medical records at both institutions. A1c levels drawn 5-7 months after the baseline value will be recorded.

Secondary outcome measures. Diabetes self-management behaviors will be assessed with the Summary of Diabetes Self-Care Activities Measure (SDSCA)[112]. This is a brief survey instrument to assess the following domains: general diet, specific (diabetes) diet, physical activity, diabetes medication use, and blood glucose monitoring. In a 2009 review of psychometric tools to assess diabetes self-management behaviors, the SDSCA was one of only three instruments to meet all appraisal criteria[113]. The SDSCA is also the most commonly used instrument for these domains worldwide, with good validity evidence in several languages, including Spanish[114,115]. Furthermore, we used this instrument with Hispanic/Latino patients during the early phase (Step 1) for this project[22]. The SDSCA will be administered by the same language-congruent study staff at each measure.

Seated blood pressure measurements (systolic and diastolic) will be made on the right arm using an automated BP Tru BPM-200 device after sitting quietly for five minutes[116]. Blood pressure will be measured three times; the average of the second and third readings will be used in statistical analyses. Most recent values may also be abstracted from the electronic medical records.

Weight will be measured to the nearest 0.1 kg using a single portable scale (Seca 880 Digital Floor Scale). Height will be measured to the nearest 0.1 cm at baseline only using a stadiometer. Participants will be asked to remove shoes prior to both measurements. Body mass index (BMI) is calculated as weight (kg)/height squared (m2). Most recent values may also be abstracted from the electronic medical records.

Total cholesterol, HDL cholesterol, and triglycerides will be measured from the same blood sample used to derive the outcome measure. LDL-cholesterol will be calculated for each participant based on these values: LDL-cholesterol=total cholesterol – HDL cholesterol – (triglycerides/5).

- Baseline LDL-cholesterol values will be drawn at the baseline study visit. LDL-cholesterol values drawn up to two weeks prior to the baseline visit date as part of usual clinical care may be used as the baseline LDL-cholesterol value. Recent values recorded as part of usual clinical care within a week prior to the study visit may be abstracted from the electronic medical records.
- Cholesterol values at 3 months will be drawn at the 3 month study visit. Recent values recorded as part of usual clinical care within two weeks prior to the visit may be abstracted from the electronic medical records.
- Subsequent cholesterol levels will not be drawn or abstracted.

The total volume of whole blood drawn from each participant will be approximately 8 mL (3 mL for hemoglobin A1C testing and 5 mL for cholesterol testing) when visiting the lab. Over-the-counter A1c Test kits will be mailed to patients unable to visit the lab. Study staff with call patients to confirm reception of kit and go through the instructions.

All study tasks can be completed in-person in the clinical setting, virtually, or a combination of both, to accommodate the growing interest of patients to perform tasks virtually, when possible. This flexibility is noted in the Study Tasks Table below.

The number of diabetes-related office visits will be abstracted from the electronic medical records and categorized as a visit with a primary care provider, endocrinologist, pharmacist, care manager, diabetes educator, dietician, community health worker or promotora. These visits will be recorded for the six-month interval before and after intervention (or control) delivery.

Theory-based measures. We will measure diabetes-related self-efficacy with the Diabetes Self-Efficacy Scale (α=0.85)[117], used in other studies with Spanish-speaking patients[118], including one from our study team that we used to generate preliminary data for this proposal[22]. We will measure diabetes-related outcome expectations with the Outcome Expectancies Questionnaire[119], and social support for diabetes with items from the Diabetes Care Profile[120], which we also used in our pilot study.

Story identification and transportation will be assessed through a 14-item scale developed by Larkey and colleagues that demonstrated very good construct validity among Hispanic, Spanish-speaking populations[121,122], with initial predictive validity on the transportation (emotional engagement) scale[84].

Intervention follow-up exposure. During follow-up measures, we will assess the number of times each participant viewed the digital storytelling videos and whether they viewed it with friends or family. This will provide an assessment of intervention dose for the intervention group following the supervised viewing at enrollment.

Table 2. Measures

Data Collected	Baseline	6 weeks	3 months	6 months
Demographics, health status, health literacy, and diabetes knowledge	X			
Hemoglobin A1c	X		X	X
Diabetes self-management behaviors	X	X	X	X
Systolic and diastolic blood pressure	X		X	
LDL-cholesterol	X		X	
Body mass index	X		X	
Diabetes-related healthcare utilization	X		X	X
Diabetes self-efficacy, outcome expectations, and social support	X	X	X	X
Story identification and transportation	X			
Intervention follow-up exposure		X	X	X

Study Coordinator Training and Quality Assurance

The Mayo Clinic project coordinator will work with site investigators and study coordinators to oversee training of bilingual study staff at each site. We have previously described our experience training study teams for intervention delivery among Hispanic populations[67]. The project coordinator will monitor quality control of the data through review of participant forms and procedures every three months. Moreover, the study statistician will perform monthly data checks (i.e., missing data, outliers).

Treatment Fidelity

Because this work will be conducted in clinical settings, it is important to evaluate barriers and facilitators to implementation across the two primary care sites[123]. We will evaluate subset domains of the intervention delivery construct as outlined by the recently reported NIH-sponsored framework for enhancing the value of research for dissemination and implementation[124].

In the design and conduct of this study, we have incorporated recommendations from the Treatment Fidelity Workgroup[125]. An intervention guide and checklist will be developed by the study team for each intervention and control component. Likewise, a checklist will be derived for collection of follow-up measures. Site principal investigators will randomly select and attend 5% of the patient visits. Based on these observations, bi-weekly meetings will be held with the study staff at each site (via videoconference) to reinforce treatment fidelity, provide corrective feedback, and to conduct additional training if needed.

Implementation adaptation. There is increasing recognition of the need for transparent reporting on adaptations of intervention implementation to fit local contexts[124,126]. The “core” component of the intervention for this study, i.e., viewing the video, will be standard across both sites. However, during the pilot study, we learned that the timing of intervention delivery (e.g., before or after office visit), associated visit type (e.g., during diabetes classes versus during provider visits), and recruitment procedures will vary slightly between sites. We will conduct semi-structured interviews with study staff and clinical staff at both sites to assess rationale for site-specific adaptations across each of these domains, and to further elucidate generalizable best practices for implementation of this intervention. These various conditions (timing, visit type) as well as study site will be tracked and assessed for any moderating effects.

Intervention reach and adoption. Intervention reach will be reported as the percentage of invited patients who choose to participate. The differential demographic and disease-specific characteristics of these two groups will be reported and compared. Intervention adoption will be reported as the percentage of eligible patients invited to participate at each site. These groups will likewise be compared. Site-specific differences in reach and adoption will be explored through interviews with staff at each site as described above.

Sample size considerations

Our preliminary data suggest that we may expect a moderate effect size for the intervention (25%-50% times the standard deviation). This is similar to two recent meta-analyses[127,128] of related work that informs the effect size we can expect to observe in the proposed study. The first meta-analysis of 11 randomized trials examined the efficacy of diabetes self-management education added to usual care among Hispanic patients in primary care settings, which produced an average hemoglobin A1c reduction of 0.25 standard deviations[127]. The second meta-analysis of 20 randomized trials evaluated the efficacy of diabetes self-management education among

racial/ethnic minority populations, which showed a similar hemoglobin A1c reduction of 0.31 standard deviations[128]. While these effects sizes are smaller than that of our pilot study, we have picked the more conservative (lower) estimate of effect size to ensure ample sample size. Hence, we can expect an effect likely to be between 0.25 and 0.31 standard deviation for the type of intervention proposed. Required sample sizes for an independent samples t-test to detect these two effect sizes, assuming 80% power, a two-tailed alternative and a 5% Type I error rate, are 252 and 145 patients per group, respectively. With 200 patients per group, we will have 80% power to detect an effect size of 0.28 standard deviations via the independent samples t-test. Accounting for approximately 12% loss to follow-up, we intend to recruit 225 patients in each group, for a total sample size of 450 participants.

Data analysis plan

Aim #1: Evaluate the efficacy of a community-derived digital storytelling intervention among Hispanic adults with poorly controlled T2D.

The primary analysis will be an independent samples t-test between the group average hemoglobin A1c values at 3 months. A two-sided alternative with a 5% type I error rate will be employed.

<u>Supplementary analysis for the primary endpoint.</u> Repeated measures modeling of the individual time point A1c values (including chart abstracted values at approximately 6 months) will allow for a more detailed investigation to the timing of the impact of the intervention by including all time points (including baseline). Profile analysis (Srivastava) will further assess for difference in overall means, time point, and treatment by time interactions. Covariates indicated above including demographics and clinical covariates will be included in the repeated measures and profile analysis models to assess the sensitivity of the primary analysis conditional on these potential concomitant (mediating) influences.

<u>Secondary analyses.</u> The analyses applied to the primary endpoint will also be applied to each of the secondary endpoints. The subscales from the SDSCA (general diet, diabetes diet, physical activity, diabetes medication use, blood glucose monitoring) and the physical measurements (blood pressure, LDL-cholesterol, BMI) will be compared between groups using t-tests and repeated measures monitoring similar to the primary endpoint. As we have a *single variable* primary endpoint defined, there will be no adjustment for comparison-wise Type I error rate. Additional analyses will be performed to identify potential effect modifiers for the primary and secondary outcomes, i.e., sex, age, socioeconomic position, acculturation, health literacy, and diabetes knowledge.

Aim #2: Explore the effect of the digital storytelling intervention on proposed theory-based mediators of change.

We will examine the correlational structure of the covariates and their impact on the intervention effect size via repeated measures models. We will include procedures suggested by MacKinnon[129,130] to assess mediation, fitting three general estimating equation models to the data. We will first estimate the intervention effect separately for the dependent variable (with regression adjustment for covariates); that model provides an estimate of the total effect of the intervention. Next, we will estimate the intervention effect for each mediator (with regression adjustment for covariates); that model provides an estimate of the effect of the intervention on the mediator. Finally, we will estimate the mediated intervention effect for the dependent variable by adjusting for the mediator (and covariates); that model provides an estimate of the unmediated (i.e., direct) intervention effect, and the intervention-adjusted effect of the mediator on the dependent variable. These analyses will be done for the primary endpoint and each of the secondary endpoints to identify any mediators of the study results.

Table 3. Study Timeline

Tasks to be completed	Year 1	Year 2	Year 3	Year 4
Recruitment, enrollment, and baseline measures				
Intervention				
Follow-up measurements				
Data analysis				
Dissemination and sustainability plan				

Dissemination and Sustainability Plan

Consistent with CBPR principles[10], dissemination of research findings will be a collaborative, multi-modal effort by both academic and community partners following the same process established with prior RHCP research efforts. Dissemination will occur at RHCP meetings, community meeting places, at academic conferences, and in peer-reviewed publications. Presentations and authorship will be shared by community, clinical and academic partners.

Sustainability of successful components of the program will be a high priority. Consistent with previous RHCP efforts, sustainability planning will occur with community members. Small group brainstorming sessions will commence even before data analysis is completed. This will be an iterative process using results from brainstorming sessions to inform subsequent discussions until a collaborative plan is in place. The intervention is intentionally designed to be sustainable and scalable. The behavioral messages contained in the stories (dietary quality, physical activity, medication adherence, glucose self-monitoring) are unlikely to loose clinical relevance over time. Once effectiveness is established, the intervention will be housed online in the public domain and available as a mobile application for use in clinical settings across the country. Furthermore, the process of participatory digital storytelling intervention development contained in this work will provide a flexible template to address other health issues among diverse subset populations. This template incorporates the digital storytelling CBPR process for message development and model factors to emphasize in selecting stories to share. Furthermore, these products may be added to other evidence-based diabetes technologies to examine effectiveness of mobile applications for diabetes self-management among diverse populations.

Potential Problems & Alternative Strategies

In addition to potential challenges elucidated above, it is expected to be challenging to implement the intervention and conduct follow-up measurements in busy clinical settings. We have prepared for this challenge by pilot testing these processes in both of the sites that will participate in this study. The pilot allowed us to adapt study implementation procedures at HCMC and MPHC. We expect unforeseen site-specific implementation challenges to arise. The communication scheme we have already developed between each of the sites will be used to brainstorm and adapt implementation strategies in the face of these new challenges.

BIBLIOGRAPHY AND REFERENCES CITED

1. Menke A, Casagrande S, Geiss L, Cowie CC. Prevalence of and Trends in Diabetes Among Adults in the United States, 1988-2012. JAMA 2015;314:1021-9.
2. Murphy SL, Xu J, Kochanek KD. Deaths: final data for 2010. Natl Vital Stat Rep 2013;61:1-117.
3. Neighbors CJ, Marquez DX, Marcus BH. Leisure-time physical activity disparities among Hispanic subgroups in the United States. Am J Public Health 2008;98:1460-4.
4. Rehm CD, Penalvo JL, Afshin A, Mozaffarian D. Dietary Intake Among US Adults, 1999-2012. JAMA 2016;315:2542-53.
5. Heisler M, Faul JD, Hayward RA, Langa KM, Blaum C, Weir D. Mechanisms for racial and ethnic disparities in glycemic control in middle-aged and older Americans in the health and retirement study. Arch Intern Med 2007;167:1853-60.
6. Levine DA, Allison JJ, Cherrington A, Richman J, Scarinci IC, Houston TK. Disparities in self-monitoring of blood glucose among low-income ethnic minority populations with diabetes, United States. Ethnicity & disease 2009;19:97-103.
7. van der Velde J, Williamson DL, Ogilvie LD. Participatory action research: practical strategies for actively engaging and maintaining participation in immigrant and refugee communities. Qualitative health research 2009;19:1293-302.
8. Johnson CE, Ali SA, Shipp MP. Building community-based participatory research partnerships with a Somali refugee community. Am J Prev Med 2009;37:S230-6.
9. Wieland ML, Weis JA, Palmer T, Goodson M, Loth S, Omer F, Abbenyi A, Krucker K, Edens K, Sia IG. Physical activity and nutrition among immigrant and refugee women: a community-based participatory research approach. Women's health issues : official publication of the Jacobs Institute of Women's Health 2012;22:e225-32.
10. Israel BA, Schulz AJ, Parker EA, Becker AB. Review of community-based research: assessing partnership approaches to improve public health. Annu Rev Public Health 1998;19:173-202.

11. Wieland ML, Weis JA, Olney MW, Aleman M, Sullivan S, Millington K, O'Hara C, Nigon JA, Sia IG. Screening for tuberculosis at an adult education center: results of a community-based participatory process. Am J Public Health 2011;101:1264-7.
12. Wieland ML, Weis JA, Hanza MM, Meiers SJ, Patten CA, Clark MM, Sloan JA, Novotny PJ, Njeru JW, Abbenyi A, Levine JA, Goodson M, Porraz Capetillo MG, Osman A, Hared A, Nigon JA, Sia IG. Healthy immigrant families: Participatory development and baseline characteristics of a community-based physical activity and nutrition intervention. Contemp Clin Trials 2015;47:22-31.
13. Hinyard LJ, Kreuter MW. Using narrative communication as a tool for health behavior change: a conceptual, theoretical, and empirical overview. Health education & behavior : the official publication of the Society for Public Health Education 2007;34:777-92.
14. Palacios JF, Salem B, Hodge FS, Albarran CR, Anaebere A, Hayes-Bautista TM. Storytelling: A Qualitative Tool to Promote Health Among Vulnerable Populations. J Transcult Nurs 2015;26:346-53.
15. Murphy ST, Frank LB, Chatterjee JS, Baezconde-Garbanati L. Narrative versus Non-narrative: The Role of Identification, Transportation and Emotion in Reducing Health Disparities. J Commun 2013;63.
16. Kreuter MW, Holmes K, Alcaraz K, Kalesan B, Rath S, Richert M, McQueen A, Caito N, Robinson L, Clark EM. Comparing narrative and informational videos to increase mammography in low-income African American women. Patient education and counseling 2010;81 Suppl:S6-14.
17. Webb Hooper M, Baker EA, Robinson RG. Efficacy of a DVD-based smoking cessation intervention for African Americans. Nicotine Tob Res 2014;16:1327-35.
18. Houston TK, Allison JJ, Sussman M, Horn W, Holt CL, Trobaugh J, Salas M, Pisu M, Cuffee YL, Larkin D, Person SD, Barton B, Kiefe CI, Hullett S. Culturally appropriate storytelling to improve blood pressure: a randomized trial. Ann Intern Med 2011;154:77-84.
19. Gubrium A. Digital storytelling: an emergent method for health promotion research and practice. Health promotion practice 2009;10:186-91.
20. Gubrium AC, Hill AL, Flicker S. A situated practice of ethics for participatory visual and digital methods in public health research and practice: a focus on digital storytelling. Am J Public Health 2014;104:1606-14.
21. Formea CM, Mohamed AA, Hassan A, Osman A, Weis JA, Sia IG, Wieland ML. Lessons learned: cultural and linguistic enhancement of surveys through community-based participatory research. Progress in community health partnerships : research, education, and action 2014;8:331-6.
22. Njeru JW, Formea CM, Osman A, Goodson M, Hared A, Capetillo GP, Nigon JA, Cha SS, Weis JA, Hanza MM, Patten CA, Sia IG, Wieland ML. Diabetes Knowledge, Attitudes and Behaviors Among Somali and Latino Immigrants. Journal of immigrant and minority health / Center for Minority Public Health 2015.
23. Njeru JW, Patten CA, Hanza MM, Brockman TA, Ridgeway JL, Weis JA, Clark MM, Goodson M, Osman A, Porraz-Capetillo G, Hared A, Myers A, Sia IG, Wieland ML. Stories for change: development of a diabetes digital storytelling intervention for refugees and immigrants to minnesota using qualitative methods. BMC Public Health 2015;15:1311.
24. Wieland ML, Njeru JW, Hanza MM, Boehm DH, Singh D, Yawn BP, Patten CA, Clark MM, Weis JA, Osman A, Goodson M, Porraz Capetillo MD, Hared A, Hasley R, Guzman-Corrales L, Sandler R, Hernandez V, Novotny PJ, Sloan JA, Sia IG. Pilot Feasibility Study of a Digital Storytelling Intervention for Immigrant and Refugee Adults With Diabetes. Diabetes Educ 2017:145721717713317.
25. Lanting LC, Joung IM, Mackenbach JP, Lamberts SW, Bootsma AH. Ethnic differences in mortality, end-stage complications, and quality of care among diabetic patients: a review.Diabetes Care 2005;28:2280-8.
26. Schneiderman N, Llabre M, Cowie CC, Barnhart J, Carnethon M, Gallo LC, Giachello AL, Heiss G, Kaplan RC, LaVange LM, Teng Y, Villa-Caballero L, Aviles-Santa ML. Prevalence of diabetes among Hispanics/Latinos from diverse backgrounds: the Hispanic Community Health Study/Study of Latinos (HCHS/SOL). Diabetes Care 2014;37:2233-9.
27. Aviles-Santa ML, Hsu LL, Arredondo M, Menke A, Werner E, Thyagarajan B, Heiss G, Teng Y, Schneiderman N, Giachello AL, Gallo LC, Talavera GA, Cowie CC. Differences in Hemoglobin A1c Between Hispanics/Latinos and Non-Hispanic Whites: An Analysis of the Hispanic Community Health Study/Study of Latinos and the 2007-2012 National Health and Nutrition Examination Survey. Diabetes Care 2016;39:1010-7.
28. Haas L, Maryniuk M, Beck J, Cox CE, Duker P, Edwards L, Fisher EB, Hanson L, Kent D, Kolb L, McLaughlin S, Orzeck E, Piette JD, Rhinehart AS, Rothman R, Sklaroff S, Tomky D, Youssef G. National standards for diabetes self-management education and support. Diabetes Care 2014;37 Suppl 1:S144-53.
29. Golden SH, Brown A, Cauley JA, Chin MH, Gary-Webb TL, Kim C, Sosa JA, Sumner AE, Anton B. Health disparities in endocrine disorders: biological, clinical, and nonclinical factors--an Endocrine Society scientific statement. The Journal of clinical endocrinology and metabolism 2012;97:E1579-639.

30. Piccolo RS, Subramanian SV, Pearce N, Florez JC, McKinlay JB. Relative Contributions of Socioeconomic, Local Environmental, Psychosocial, Lifestyle/Behavioral, Biophysiological, and Ancestral Factors to Racial/Ethnic Disparities in Type 2 Diabetes. Diabetes Care 2016;39:1208-17.
31. Daviglus ML, Talavera GA, Aviles-Santa ML, Allison M, Cai J, Criqui MH, Gellman M, Giachello AL, Gouskova N, Kaplan RC, LaVange L, Penedo F, Perreira K, Pirzada A, Schneiderman N, Wassertheil-Smoller S, Sorlie PD, Stamler J. Prevalence of major cardiovascular risk factors and cardiovascular diseases among Hispanic/Latino individuals of diverse backgrounds in the United States. JAMA 2012;308:1775-84.
32. Anderson C, Zhao H, Daniel CR, Hromi-Fiedler A, Dong Q, Elhor Gbito KY, Wu X, Chow WH. Acculturation and Diabetes Risk in the Mexican American Mano a Mano Cohort. Am J Public Health 2016;106:547-9.
33. Ayala GX, Baquero B, Klinger S. A systematic review of the relationship between acculturation and diet among Latinos in the United States: implications for future research. J Am Diet Assoc 2008;108:1330-44.
34. Mainous AG, 3rd, Majeed A, Koopman RJ, Baker R, Everett CJ, Tilley BC, Diaz VA. Acculturation and diabetes among Hispanics: evidence from the 1999-2002 National Health and Nutrition Examination Survey. Public health reports 2006;121:60-6.
35. Lara M, Gamboa C, Kahramanian MI, Morales LS, Bautista DE. Acculturation and Latino health in the United States: a review of the literature and its sociopolitical context. Annu Rev Public Health 2005;26:367-97.
36. Osborn CY, Cavanaugh K, Wallston KA, Kripalani S, Elasy TA, Rothman RL, White RO. Health literacy explains racial disparities in diabetes medication adherence. J Health Commun 2011;16 Suppl 3:268-78.
37. Malmusi D, Borrell C, Benach J. Migration-related health inequalities: showing the complex interactions between gender, social class and place of origin. Social science & medicine 2010;71:1610-9.
38. Vega WA, Rodriguez MA, Gruskin E. Health disparities in the Latino population. Epidemiol Rev 2009;31:99-112.
39. Horowitz CR, Robinson M, Seifer S. Community-based participatory research from the margin to the mainstream: are researchers prepared? Circulation 2009;119:2633-42.
40. Israel BA, Schulz AJ, Parker EA, Becker AB. Review of community-based research: assessing partnership approaches to improve public health. Annu Rev Public Health 1998;19:173-202.
41. Larkey LK, Lopez AM, Minnal A, Gonzalez J. Storytelling for promoting colorectal cancer screening among underserved Latina women: a randomized pilot study. Cancer Control 2009;16:79-87.
42. Larkey LK, Gonzalez J. Storytelling for promoting colorectal cancer prevention and early detection among Latinos. Patient education and counseling 2007;67:272-8.
43. Moran MB, Murphy ST, Frank L, Baezconde-Garbanati L. The Ability of Narrative Communication to Address Health-related Social Norms. Int Rev Soc Res 2013;3:131-49.
44. Lee JK, Hecht ML, Miller-Day M, Elek E. Evaluating Mediated Perception of Narrative Health Messages: The Perception of Narrative Performance Scale. Commun Methods Meas 2011;5:126-45.
45. Slater MDR, D. Entertainment-education and elaboration likelihood: Understanding the processing of narrative persuasion. Communication Theory 2002;12:173-91.
46. Hodge FS, Pasqua A, Marquez CA, Geishirt-Cantrell B. Utilizing traditional storytelling to promote wellness in American Indian communities. J Transcult Nurs 2002;13:6-11.
47. Hoybye MT, Johansen C, Tjornhoj-Thomsen T. Online interaction. Effects of storytelling in an internet breast cancer support group. Psychooncology 2005;14:211-20.
48. Green MC, Brock TC. The role of transportation in the persuasiveness of public narratives. J Pers Soc Psychol 2000;79:701-21.
49. Strickland CJ, Squeoch MD, Chrisman NJ. Health promotion in cervical cancer prevention among the Yakama Indian women of the Wa'Shat Longhouse. J Transcult Nurs 1999;10:190-6.
50. Slater MD, Buller DB, Waters E, Archibeque M, LeBlanc M. A test of conversational and testimonial messages versus didactic presentations of nutrition information. Journal of nutrition education and behavior 2003;35:255-9.
51. Houston TK, Cherrington A, Coley HL, Robinson KM, Trobaugh JA, Williams JH, Foster PH, Ford DE, Gerber BS, Shewchuk RM, Allison JJ. The art and science of patient storytelling-harnessing narrative communication for behavioral interventions: the ACCE project. J Health Commun 2011;16:686-97.
52. Greenhalgh T, Campbell-Richards D, Vijayaraghavan S, Collard A, Malik F, Griffin M, Morris J, Claydon A, Macfarlane F. New models of self-management education for minority ethnic groups: pilot randomized trial of a story-sharing intervention. J Health Serv Res Policy 2011;16:28-36.
53. Wilson E, Chen AH, Grumbach K, Wang F, Fernandez A. Effects of limited English proficiency and physician language on health care comprehension. J Gen Intern Med 2005;20:800-6.

54. Cueva M, Kuhnley R, Revels LJ, Cueva K, Dignan M, Lanier AP. Bridging storytelling traditions with digital technology. Int J Circumpolar Health 2013;72.
55. Armistead SG. Pan-hispanic oral tradition. Oral Tradition 2004;18.
56. Larkey LK, McClain D, Roe DJ, Hector RD, Lopez AM, Sillanpaa B, Gonzalez J. Randomized Controlled Trial of Storytelling Compared to a Personal Risk Tool Intervention on Colorectal Cancer Screening in Low-Income Patients. American journal of health promotion : AJHP 2015.
57. Campbell T, Dunt D, Fitzgerald JL, Gordon I. The impact of patient narratives on self-efficacy and self-care in Australians with type 2 diabetes:stage 1 results of a randomized trial.Health Promot Int 2015;30:438-48.
58. Gubrium AC, Krause EL, Jernigan K. Strategic Authenticity and Voice: New Ways of Seeing and Being Seen as Young Mothers through Digital Storytelling. Sex Res Social Policy 2014;11:337-47.
59. Toussaint DW, Villagrana M, Mora-Torres H, de Leon M, Haughey MH. Personal stories: voices of Latino youth health advocates in a diabetes prevention initiative. Progress in community health partnerships : research, education, and action 2011;5:313-6.
60. Wyatt TH, Hauenstein EJ. Pilot testing Okay With Asthma: an online asthma intervention for school-age children. J Sch Nurs 2008;24:145-50.
61. Jernigan VB, Salvatore AL, Styne DM, Winkleby M. Addressing food insecurity in a Native American reservation using community-based participatory research. Health Educ Res 2012;27:645-55.
62. Cueva M, Kuhnley R, Revels L, Schoenberg NE, Dignan M. Digital storytelling: a tool for health promotion and cancer awareness in rural Alaskan communities. Int J Circumpolar Health 2015;74:28781.
63. Montague E, Perchonok J. Health and wellness technology use by historically underserved health consumers: systematic review. J Med Internet Res 2012;14:e78.
64. Rochester Healthy Community Partnership. (Accessed September 3, 2016, www.rochesterhealthy.org.)
65. Hawley NC, Wieland ML, Weis JA, Sia IG. Perceived impact of human subjects protection training on community partners in community-based participatory research. Progress in community health partnerships : research, education, and action 2014;8:241-8.
66. Amico KL, Wieland ML, Weis JA, Sullivan SM, Nigon JA, Sia IG. Capacity building through focus group training in community-based participatory research. Educ Health (Abingdon) 2011;24:638.
67. Bronars CA, Hanza MM, Meiers SJ, Patten CA, Clark MM, Nigon JA, Weis JA, Wieland ML, Sia IG. Treatment Fidelity Among Family Health Promoters Delivering a Physical Activity and Nutrition Intervention to Immigrant and Refugee Families. Health Educ Behav 2017;44:262-70.
68. Hanza MM, Wieland ML, Weis JA, Meiers SJ, Patten CA, Clark MM, Sloan JA, Novotny PJ, Njeru JW, Abbenyi A, Levine JA, Goodson M, Porraz Capetillo MG, Osman A, Hared A, Nigon JA, Sia IG. Healthy Immigrant Families: Utilizing a CBPR approach to develop, implement and evaluate a community-based physical activity and nutrition intervention for immigrants and refugees. In: 8th Annual Conference on the Science of Dissemination and Implementation 2015; Washington DC; 2015.
69. Goel MS, McCarthy EP, Phillips RS, Wee CC. Obesity among US immigrant subgroups by duration of residence. JAMA 2004;292:2860-7.
70. Wieland ML, Tiedje K, Meiers SJ, Mohamed AA, Formea CM, Ridgeway JL, Asiedu GB, Boyum G, Weis JA, Nigon JA, Patten CA, Sia IG. Perspectives on physical activity among immigrants and refugees to a small urban community in Minnesota. Journal of immigrant and minority health / Center for Minority Public Health 2015;17:263-75.
71. Tiedje K, Wieland ML, Meiers SJ, Mohamed AA, Formea CM, Ridgeway JL, Asiedu GB, Boyum G, Weis JA, Nigon JA, Patten CA, Sia IG. A focus group study of healthy eating knowledge, practices, and barriers among adult and adolescent immigrants and refugees in the United States. The international journal of behavioral nutrition and physical activity 2014;11:63.
72. Mohamed AA, Hassan AM, Weis JA, Sia IG, Wieland ML. Physical activity among Somali men in Minnesota: barriers, facilitators, and recommendations. Am J Mens Health 2014;8:35-44.
73. Wieland ML, Weis JA, Yawn BP, Sullivan SM, Millington KL, Smith CM, Bertram S, Nigon JA, Sia IG. Perceptions of tuberculosis among immigrants and refugees at an adult education center: a community-based participatory research approach. Journal of immigrant and minority health / Center for Minority Public Health 2012;14:14-22.
74. Wieland ML, Nelson J, Palmer T, O'Hara C, Weis JA, Nigon JA, Sia IG. Evaluation of a tuberculosis education video among immigrants and refugees at an adult education center: a community-based participatory approach. J Health Commun 2013;18:343-53.
75. Rochester Healthy Community Partnership. (Accessed August 9, 2016, at http://cpr.unm.edu/research-projects/cbpr-project/cbpr-model-additional-language.html.)

76. Wilhelm AK, Jacobson DJ, Guzman-Corrales L, Fan C, Baker K, Njeru JW, Wieland ML, Boehm DH. Regional Variation in Diabetic Outcomes by Country-of-Origin and Language in an Urban Safety Net Hospital. J Community Health 2016.
77. Wieland ML, Morrison TB, Cha SS, Rahman AS, Chaudhry R. Diabetes care among Somali immigrants and refugees. J Community Health 2012;37:680-4.
78. Njeru JW, Tan EM, St Sauver J, Jacobson DJ, Agunwamba AA, Wilson PM, Rutten LJ, Damodaran S, Wieland ML. High Rates of Diabetes Mellitus, Pre-diabetes and Obesity Among Somali Immigrants and Refugees in Minnesota: A Retrospective Chart Review. Journal of immigrant and minority health / Center for Minority Public Health 2015.
79. Patten CA, Windsor RA, Renner CC, Enoch C, Hochreiter A, Nevak C, Smith CA, Decker PA, Bonnema S, Hughes CA, Brockman T. Feasibility of a tobacco cessation intervention for pregnant Alaska Native women. Nicotine Tob Res 2010;12:79-87.
80. Patten CA, Enoch C, Renner CC, Larsen K, Decker PA, Anderson KJ, Nevak C, Glasheen A, Offord KP, Lanier A. Evaluation of a Tobacco Educational Intervention for Pregnant Alaska Native Women. J Health Dispar Res Pract 2008;2:33-50.
81. Patten CA, Offord KP, Ames SC, Decker PA, Croghan IT, Dornelas EA, Pingree S, Boberg EW, Gustafson DH, Ahluwalia JS, Wolter TD, Hurt RD. Differences in adolescent smoker and nonsmoker perceptions of strategies that would help an adolescent quit smoking. Ann Behav Med 2003;26:124-33.
82. Patten CA, Offord KP, Hurt RD, Sanderson Cox L, Thomas JL, Quigg SM, Croghan IT, Wolter TD, Decker PA. Training support persons to help smokers quit: a pilot study. Am J Prev Med 2004;26:386-90.
83. Patten CA, Vickers KS, Martin JE, Williams CD. Exercise interventions for smokers with a history of alcoholism: exercise adherence rates and effect of depression on adherence. Addict Behav 2003;28:657-67.
84. Larkey LK, McClain D, Roe DJ, Hector RD, Lopez AM, Sillanpaa B, Gonzalez J. Randomized Controlled Trial of Storytelling Compared to a Personal Risk Tool Intervention on Colorectal Cancer Screening in Low-Income Patients. American journal of health promotion : AJHP 2015;30:e59-70.
85. Clark MM, Abrams DB, Niaura RS, Eaton CA, Rossi JS. Self-efficacy in weight management. Journal of consulting and clinical psychology 1991;59:739-44.
86. Clark MM, Bradley KL, Jenkins SM, Mettler EA, Larson BG, Preston HR, Liesinger JT, Werneburg BL, Hagen PT, Harris AM, Riley BA, Olsen KD, Vickers Douglas KS. Improvements in Health Behaviors, Eating Self-Efficacy, and Goal-Setting Skills Following Participation in Wellness Coaching. American journal of health promotion : AJHP 2015.
87. Singh JA, Satele D, Pattabasavaiah S, Buckner JC, Sloan JA. Normative data and clinically significant effect sizes for single-item numerical linear analogue self-assessment (LASA) scales. Health Qual Life Outcomes 2014;12:187.
88. Shaibi G, Singh D, De Filippis E, Hernandez V, Rosenfeld B, Otu E, Montes de Oca G, Levey S, Radecki Breitkopf C, Sharp R, Olson J, Cerhan J, Thibodeau S, Winkler E, Mandarino L. The Sangre Por Salud Biobank: Facilitating Genetic Research in an Underrepresented Latino Community. Public Health Genomics 2016;19:229-38.
89. LeBlanc A, Herrin J, Williams MD, Inselman JW, Branda ME, Shah ND, Heim EM, Dick SR, Linzer M, Boehm DH, Dall-Winther KM, Matthews MR, Yost KJ, Shepel KK, Montori VM. Shared Decision Making for Antidepressants in Primary Care: A Cluster Randomized Trial. JAMA Intern Med 2015;175:1761-70.
90. US Department of Health and Human Services. Making health communication programs work. In. Washington DC: National Institutes of Health, National Cancer Institute; 2004.
91. Lancaster GA, Dodd S, Williamson PR. Design and analysis of pilot studies: recommendations for good practice. Journal of evaluation in clinical practice 2004;10:307-12.
92. Rounsaville BJ, Carrol, K. M., Onken, L.S. A stage model of behavioral therapies research: getting started and moving on from stage 1. Clinical Psychology: Science and Practice 2001;8:133-42.
93. Bandura A. Health promotion by social cognitive means. Health Educ Behav 2004;31:143-64.
94. Greenhalgh T, Collard A, Campbell-Richards D, Vijayaraghavan S, Malik F, Morris J, Claydon A. Storylines of self-management: narratives of people with diabetes from a multiethnic inner city population. J Health Serv Res Policy 2011;16:37-43.
95. Sarkar U, Fisher L, Schillinger D. Is self-efficacy associated with diabetes self-management across race/ethnicity and health literacy? Diabetes Care 2006;29:823-9.
96. Bernal H, Woolley S, Schensul JJ, Dickinson JK. Correlates of self-efficacy in diabetes self-care among Hispanic adults with diabetes. Diabetes Educ 2000;26:673-80.

97. Strizich G, Kaplan RC, Gonzalez HM, Daviglus ML, Giachello AL, Teng Y, Lipton RB, Grober E. Glycemic control, cognitive function, and family support among middle-aged and older Hispanics with diabetes: The Hispanic Community Health Study/Study of Latinos. Diabetes Res Clin Pract 2016;117:64-73.
98. Standards of medical care in diabetes--2015: summary of revisions. Diabetes Care 2015;38 Suppl:S4.
99. Core Quality Measures. 2016. (Accessed February 23, 2016, at https://www.cms.gov/Medicare/Quality-Initiatives-Patient-Assessment-Instruments/QualityMeasures/Core-Measures.html.)
100. The D5 for Diabetes. 2016. (Accessed at http://mncm.org/reports-and-websites/the-d5/.)
101. Borrelli B, Sepinwall D, Ernst D, Bellg AJ, Czajkowski S, Breger R, DeFrancesco C, Levesque C, Sharp DL, Ogedegbe G, Resnick B, Orwig D. A new tool to assess treatment fidelity and evaluation of treatment fidelity across 10 years of health behavior research. Journal of consulting and clinical psychology 2005;73:852-60.
102. Glasgow RE, Lichtenstein E, Marcus AC. Why don't we see more translation of health promotion research to practice? Rethinking the efficacy-to-effectiveness transition. Am J Public Health 2003;93:1261-7.
103. Lorig KR, Sobel DS, Stewart AL, Brown BW, Jr., Bandura A, Ritter P, Gonzalez VM, Laurent DD, Holman HR. Evidence suggesting that a chronic disease self-management program can improve health status while reducing hospitalization: a randomized trial. Medical care 1999;37:5-14.
104. Peimani M, Rambod C, Omidvar M, Larijani B, Ghodssi-Ghassemabadi R, Tootee A, Esfahani EN. Effectiveness of short message service-based intervention (SMS) on self-care in type 2 diabetes: A feasibility study. Prim Care Diabetes 2015.
105. Piette JD, List J, Rana GK, Townsend W, Striplin D, Heisler M. Mobile Health Devices as Tools for Worldwide Cardiovascular Risk Reduction and Disease Management. Circulation 2015;132:2012-27.
106. de Jongh T, Gurol-Urganci I, Vodopivec-Jamsek V, Car J, Atun R. Mobile phone messaging for facilitating self-management of long-term illnesses. Cochrane Database Syst Rev 2012;12:CD007459.
107. Flores G, Abreu M, Tomany-Korman SC. Limited english proficiency, primary language at home, and disparities in children's health care: how language barriers are measured matters. Public Health Rep 2005;120:418-30.
108. Lee SY, Stucky BD, Lee JY, Rozier RG, Bender DE. Short Assessment of Health Literacy-Spanish and English: a comparable test of health literacy for Spanish and English speakers. Health services research 2010;45:1105-20.
109. Garcia AA, Villagomez ET, Brown SA, Kouzekanani K, Hanis CL. The Starr County Diabetes Education Study: development of the Spanish-language diabetes knowledge questionnaire.Diabetes Care 2001;24:16-21.
110. Laiteerapong N, Fairchild PC, Chou CH, Chin MH, Huang ES. Revisiting Disparities in Quality of Care Among US Adults With Diabetes in the Era of Individualized Care, NHANES 2007-2010. Medical care 2015;53:25-31.
111. Diabetes HbA1c 2016.(Accessed Sep 8, 2016, hrsa.gov/quality/toolbox/measures/diabetes/index.html.)
112. Toobert DJ, Hampson SE, Glasgow RE. The summary of diabetes self-care activities measure: results from 7 studies and a revised scale. Diabetes Care 2000;23:943-50.
113. Eigenmann CA, Colagiuri R, Skinner TC, Trevena L. Are current psychometric tools suitable for measuring outcomes of diabetes education? Diabet Med 2009;26:425-36.
114. Caro-Bautista J, Morilla-Herrera JC, Villa-Estrada F, Cuevas-Fernandez-Gallego M, Lupianez-Perez I, Morales-Asencio JM. [Spanish cultural adaptation and psychometric validation of the Summary of Diabetes Self-Care Activities measure (SDSCA) among persons with type 2 diabetes mellitus]. Aten Primaria 2015.
115. Vincent D, McEwen MM, Pasvogel A. The validity and reliability of a Spanish version of the summary of diabetes self-care activities questionnaire. Nursing research 2008;57:101-6.
116. Chobanian AV, Bakris GL, Black HR, Cushman WC, Green LA, Izzo JL, Jr., Jones DW, Materson BJ, Oparil S, Wright JT, Jr., Roccella EJ. The Seventh Report of the Joint National Committee on Prevention, Detection, Evaluation, and Treatment of High Blood Pressure: the JNC 7 report. Jama 2003;289:2560-72.
117. Lorig K, Stewart, A., Ritter, P., González, V., Laurent, D., Lynch, J. Outcome Measures for Health Education and Other Health Care Interventions. Thousand Oaks, CA: Sage; 1996.
118. Lorig K, Ritter PL, Villa FJ, Armas J. Community-based peer-led diabetes self-management: a randomized trial. Diabetes Educ 2009;35:641-51.
119. Glasgow RE, Toobert DJ, Riddle M, Donnelly J, Mitchell DL, Calder D. Diabetes-specific social learning variables and self-care behaviors among persons with type II diabetes. Health Psychol 1989;8:285-303.
120. Fitzgerald JT, Davis WK, Connell CM, Hess GE, Funnell MM, Hiss RG. Development and validation of the Diabetes Care Profile. Eval Health Prof 1996;19:208-30.

121. Larkey LK, Lopez AM, Roe DJ. Measures to assess narrative influences on cancer prevention behaviors. In: American Association for Cancer Research: Frontiers in Cancer Prevention Research. Washington, D.C.; 2008.
122. Kim S, Shin CN, Larkey LK. Development and validation of measures of narrative factor effects on health behavior change. Under Review 2016.
123. Glasgow RE, Vinson C, Chambers D, Khoury MJ, Kaplan RM, Hunter C. National Institutes of Health approaches to dissemination and implementation science: current and future directions. Am J Public Health 2012;102:1274-81.
124. Neta G, Glasgow RE, Carpenter CR, Grimshaw JM, Rabin BA, Fernandez ME, Brownson RC. A Framework for Enhancing the Value of Research for Dissemination and Implementation. Am J Public Health 2015;105:49-57.
125. Bellg AJ, Borrelli B, Resnick B, Hecht J, Minicucci DS, Ory M, Ogedegbe G, Orwig D, Ernst D, Czajkowski S. Enhancing treatment fidelity in health behavior change studies: best practices and recommendations from the NIH Behavior Change Consortium. Health psychology : official journal of the Division of Health Psychology, American Psychological Association 2004;23:443-51.
126. Hawe P, Shiell A, Riley T. Complex interventions: how "out of control" can a randomised controlled trial be? BMJ 2004;328:1561-3.
127. Ferguson S, Swan M, Smaldone A. Does diabetes self-management education in conjunction with primary care improve glycemic control in Hispanic patients? A systematic review and meta-analysis. Diabetes Educ 2015;41:472-84.
128. Ricci-Cabello I, Ruiz-Perez I, Rojas-Garcia A, Pastor G, Rodriguez-Barranco M, Goncalves DC. Characteristics and effectiveness of diabetes self-management educational programs targeted to racial/ethnic minority groups: a systematic review, meta-analysis and meta-regression. BMC Endocr Disord 2014;14:60.
129. MacKinnon DP, Fairchild AJ, Fritz MS. Mediation analysis. Annu Rev Psychol 2007;58:593-614.
130. MacKinnon DP. Introduction to statistical mediation analysis. New York, NY: Taylor & Francis Group, LLC; 2008.
