# Supplementary material for: Digital Storytelling Intervention for Hemoglobin A1c Control Among Hispanic Adults With Type 2 Diabetes: A Randomized Clinical Trial
Source: JAMA Netw Open. 2024 Aug 2;7(8):e2424781. doi: 10.1001/jamanetworkopen.2024.24781 (PMC11297376; doi:10.1001/jamanetworkopen.2024.24781)
Supplement: Supplement 3. — Data Sharing Statement [file jamanetwopen-e2424781-s003.pdf]

Data Sharing Statement

Wieland. Digital Storytelling Intervention for Hemoglobin A_{1c} Control Among Hispanic Adults With Type 2 Diabetes. *JAMA Netw Open*. Published August 02, 2024.
doi:10.1001/jamanetworkopen.2024.24781

Data
Data available: Yes
Data types: Deidentified participant data
How to access data: The datasets generated during and/or analyzed in the current study are available from the corresponding author upon reasonable request, pending approval from community partners: wieland.mark@mayo.edu
When available: With publication

Supporting Documents
Document types: None

Additional Information
Who can access the data: The datasets generated during and/or analyzed in the current study are available from the corresponding author upon reasonable request, pending approval from community partners.
Types of analyses: For any purpose.
Mechanisms of data availability: With investigator support.
